# Inhibition of Protein arginine methyltransferase 6 reduces reactive oxygen species production and attenuates aminoglycoside- and cisplatin-induced hair cell death

**DOI:** 10.7150/thno.37362

**Published:** 2020-01-01

**Authors:** Yingzi He, Wen Li, Zhiwei Zheng, Liping Zhao, Wenyan Li, Yunfeng Wang, Huawei Li

**Affiliations:** 1 Department of ENT institute and Otorhinolaryngology, Eye & ENT Hospital, State Key Laboratory of Medical Neurobiology, NHC Key Laboratory of Hearing Medicine Research, Fudan University, Shanghai, 200032, PR China; 2 Department of Otorhinolaryngology Head and Neck Surgery, The First Affiliated Hospital, School of Medicine, Xiamen University, Xiamen, 361003, China; 3 Institutes of Biomedical Sciences, Fudan University, Shanghai, 200032, PR China; 4 Shanghai Engineering Research Centre of Cochlear Implant, Shanghai, 200031, PR China; 5 The Institutes of Brain Science and the Collaborative Innovation Center for Brain Science, Fudan University, Shanghai, 200032, China

**Keywords:** Aminoglycoside, cisplatin, ototoxicity, mitochondrial dysfunction, hair cell, protectio

## Abstract

Hair cells in the inner ear have been shown to be susceptible to ototoxicity from some beneficial pharmaceutical drugs, such as aminoglycosides and cisplatin. Thus, there is great interest in discovering new targets or compounds that protect hair cells from these ototoxic drugs. Epigenetic regulation is closely related to inner ear development; however, little is known about epigenetic regulation in the process of ototoxic drugs-induced hearing loss.

**Methods**: In this study, we investigated the role of protein arginine methyltransferase 6 (PRMT6) in aminoglycoside- and cisplatin-induced hair cell loss by using EPZ020411, a selective small molecule PRMT6 inhibitor, *in vitro* in neonatal mouse cochlear explants and* in vivo* in C57BL/6 mice. We also took advantage of the HEI-OC1 cell line to evaluate the anti-apoptosis effects of PRMT6 knockdown on cisplatin-induced ototoxicity. Apoptotic cells were identified using cleaved caspase-3 staining and TUNEL assay. The levels of reactive oxygen species (ROS) were evaluated by DCFH-DA and cellROX green staining. The mitochondrial membrane potential (ΔΨm) were determined by JC-1, TMRM, and rhodamine 123 staining.

**Results**: We found that EPZ020411 significantly alleviated neomycin- and cisplatin-induced cell apoptosis and increased hair cell survival. Moreover, pretreatment with EPZ020411 could attenuate neomycin- and cisplatin-induced hearing loss *in vivo*. Mechanistic studies revealed that inhibition of PRMT6 could reverse the increased expression of caspase-3 and cytochrome *c* translocation, mitochondrial dysfunction, increased accumulation of ROS, and activation of cell apoptosis after cisplatin injury.

**Conclusions**: Our findings suggested that PRMT6 might serve as a new therapeutic target to prevent hearing loss caused by aminoglycoside- and cisplatin-induced ototoxicity by preventing ROS formation and modulating the mitochondria-related damage and apoptosis.

## Introduction

Hearing loss can be caused by a variety of factors, including excessive noise, ototoxic pharmaceutical agents, genetic disorders, and aging. Ototoxicity is a serious toxic side effect of several beneficial pharmaceutical drugs including aminoglycoside antibiotics such as neomycin and platinum-based chemotherapy agents such as cisplatin 1. Ototoxic drugs can cause irreversible damage to the hair cells within the inner ear leading to hearing loss and balance disorders. Unfortunately, there are currently no clinically approved and effective agents that can prevent the ototoxic side effects of these valuable therapeutic drugs. Protection against ototoxicity is thus an important issue that has attracted considerable attention regarding our understanding of the molecular and biochemical mechanisms underlying hair cell damage, and much effort in the field has focused on identifying new targets or compounds that can attenuate or prevent the toxic side effects of these otherwise beneficial pharmaceutical drugs.

Epigenetic modifications, which refer to the modulation of gene expression by post-translational modification of the chromatin structure without involving alterations in the DNA sequence, have significant roles in diverse biological and cellular processes 2-4. Recently, significant progress has been made in the auditory field in understanding the functions of epigenetic modifications in transcriptional regulation. It is important to point out that epigenetics, especially methylation, have been found to occur throughout all processes of inner ear morphogenesis, including otic induction, patterning, and hair cell differentiation 5-11, and mutations in epigenetic factors have been shown to be associated with sensorineural hearing loss in humans 12, 13. In addition, histone-modifying enzymes have emerged as critical regulators of hair cell survival and supporting cell proliferation in fish and birds 14-16. Thus, understanding the epigenetic mechanisms in ototoxic hearing loss and developing drugs to maintain proper epigenetic regulation might provide a way to reverse abnormal gene expression profiles caused by ototoxic insults and thus prevent hair cell death.

Arginine methylation is an abundant posttranslational modification found in eukaryotic organisms and is catalyzed by the protein arginine methyltransferases (PRMTs) 17. PRMTs play critical roles in numerous biological pathways by regulating protein-protein interactions either positively or negatively depending on the modification status and the site of modification 18, 19. PRMT6 is a nuclear enzyme that primarily catalyzes the asymmetric dimethylation of histone H3 at arginine 2 (H3R2me2a) 20. PRMT6 has been implicated in a number of basic cellular processes, including regulation of the cell cycle 21, nuclear receptor-mediated transcription 22, and maintenance of stem cell pluripotency 23. PRMT6 overexpression has been reported recently in several types of cancers, including melanoma 24 and bladder and lung cancers 25, suggesting that inhibition of PRMT6 might be a promising new strategy for therapeutic intervention. However, no experimental evidence supports a role for PRMT6 in the inner ear, particularly in regulating the survival of hair cells. A small molecule PRMT6 inhibitor, EPZ020411, was recently identified 26, and this inhibitor is selective for PRMT6 over other methyltransferases. Studies in rats have shown good bioavailability of EPZ020411 following subcutaneous dosing making it a suitable molecule for potential *in vivo* studies 26.

In this study, we showed that inhibition of PRMT6 by EPZ020411 decreases the cells' sensitivity to aminoglycoside and cisplatin toxicity. Mechanistically, we revealed that PRMT6 inhibition using siRNA promotes the survival of hair cells by altering mitochondrial dysfunction and decreasing ROS accumulation.

## Materials and methods

### Postnatal cochlear explants and drug administration

All experiments were approved by the Shanghai Medical Experimental Animal Administrative Committee. Cochleae from C57BL/6 mice at postnatal day (P) 2 were dissected and cleaned of surrounding tissue and bone in phosphate buffered saline (PBS). The cochlear explants were stuck to a glass coverslip coated with Cell-Tak (BD Biosciences, Franklin Lakes, NJ, USA). Explants were incubated in DMEM/F12 medium supplemented with N2/B27 (Invitrogen) and ampicillin at 37°C in a 5% CO_2_/95% air atmosphere overnight prior to each treatment in order to stabilize the explants. EPZ020411 was purchased from Selleck Chemicals (Houston, TX, USA, S7820) and dissolved at a stock concentration of 10 mM and further diluted to the desired concentrations (20 μM and 40 μM). Neomycin sulfate (0.5 mM and 1 mM; Sigma-Aldrich, St. Louis, MO, USA, N6386) and cisplatin (20 μM; Sigma, 47930) were used to damage hair cells.

### HEI-OC1 cell culture

HEI-OC1 cells were grown under permissive conditions (33°C, 10% CO2) in high-glucose Dulbecco's Modified Eagle's Medium (DMEM; Gibco BRL, Gaithersburg, MD, USA) containing 10% fetal bovine serum (FBS; Gibco BRL) without antibiotics. The cells were subcultured at 80% confluence using 0.25% trypsin/EDTA (Life Technologies, 25200056).

### Neomycin treatment *in vivo*

Wild-type C57BL/6 mice were used for all *in vivo* studies. The 5-day-old mice were undergone low temperature anesthesia. Briefly, the mice were kept on 4℃ for 10 min for inducing short-term anesthesia and rapid recovery. A retro-auricular surgical approach was used in 5-day-old mice following anesthesia. To assess the protective effect of EPZ020411 on chronic models of ototoxicity, the left ears of the mice were pretreated with EPZ020411 at 10 mM for 1 μl once, while the contralateral (right) ears were treated with sterile saline. Two days after administration of the drug, neomycin was injected subcutaneously once per day for five consecutive days. The neomycin was dissolved in sterile saline at 20 mg/ml so that a final dose of 200 mg of neomycin/kg of body weight was obtained by injecting 0.01 ml/g of body weight. The detailed protocol for neomycin administration was given previously 27. The hearing threshold was evaluated by ABR measurement at P28.

To test the protective effect of EPZ020411 on acute models of ototoxicity, each animal received a single intraperitoneally (i.p.) injection of 10 mM EPZ020411 for 10 mg/kg, while the controls were injected with sterile saline. Two hours later, 100 mg/kg neomycin was administered through i.p. injection at P28 followed 30 min later by a single dose of 300 mg/kg furosemide. The hearing threshold was evaluated by ABR measurement two days later (P30).

### Cisplatin treatment *in vivo*

We used 30 mg/kg cisplatin (1 mg/ml stock in sterile saline [0.9% NaCl]) through i.p. injection at P28 based on previous reports 28. Briefly, the mice received 1 ml pre-warmed saline by subcutaneous injection 1 day before cisplatin injection. To test the protective effect of EPZ020411, EPZ020411 was injected trans-tympanically at 10 mM for 5 μl into the left ears, while the contralateral (right) ears were injected with the same volume of sterile saline and were used as the controls. Two hours later, 30 mg/kg cisplatin at a concentration of 1 mg/ml was administered by i.p. injection. After cisplatin administration, 1 ml of pre-warmed saline was injected subcutaneously twice a day for 7 consecutive days to ameliorate dehydration and kidney toxicity, and ABR threshold shifts were measured at indicated time points after cisplatin injection.

### siRNA transfection in HEI-OC-1 cells

Three PRMT6-specific mouse siRNAs were designed to knock down the expression of PRMT6 in HEI-OC-1 cells. An siRNA encoding a nonsense sequence was designed as the negative control. HEI-OC-1 cells were transfected with PRMT6-siRNA or negative-siRNA according to the manufacturer's instructions. Briefly, HEI-OC-1 cells were plated in 6-well culture plates at density of 3 × 105 and transfected with siRNA by using Lipofectamine 2000 reagent (Invitrogen, Waltham, USA). Briefly, for each well, 5 μl Lipofectamine 2000 was diluted in 250 μl Opti-MEMI medium (Thermo Fisher Scientific). This mixture was carefully added to a solution containing 200 nM siRNA in 250 μl Opti-MEMI medium. The solution was incubated for 20 min at room temperature, and then gently dripped into the HEI-OC-1 cells in 1.5 ml antibiotic free medium. The Opti-MEM medium was replaced 6 later with DMEM containing fetal bovine serum. Twenty-four hour after transfections, HEI-OC-1 cells were treated with cisplatin for 24 h and then collected for immunofluorescence and flow cytometry assays. The following siRNA was used to knock down the expression of PRMT6: PRMT6 siRNA-01, sense 5′-GAGGAAAAGACCAAAGACUTT-3′ and antisense: 5′-AGUCUUUGGUCUUUUCCUCAT-3′; PRMT6 siRNA-02, sense: 5′-GGACUGAUGCAGUGUGCUUTT-3′ and antisense: 5′-AAGCACACUGCAUCAGUCCTT-3′; PRMT6 siRNA-03, sense: 5′-CCUGGAAAGCAUGUAGUAUTT-3′ and antisense: 5′- AUACUACAUGCUUUCCAGGTT3′; negative control, sense: GUGAGCGUCUAUAUACCAUTT and antisense: AUGGUAUAUAGACGCUCACTT.

### Immunofluorescence

Samples fixed in 4 % paraformaldehyde were rinsed three times with PBS and permeabilized with 1% Triton X-100 in PBS (PBST) for 30 min at room temperature. Permeabilized samples were then blocked with 10% donkey serum in PBST for 1 h followed by incubation with the primary antibodies overnight at 4°C. The primary antibodies used in the present study were anti-myosin 7a antibody (1:500 dilution; Proteus Biosciences, Ramona, CA, USA, 25-6790), anti-parvalbumin antibody (1:500 dilution; Abcam, Cambridge, MA, USA, 32895), anti-PRMT6 antibody (1:500 dilution; Abcam, 104834), cytochrome c (1:200 dilution; Cell Signaling Technology, Inc., Danvers, MA, USA, 4272), and anti-cleaved caspase-3 antibody (1:200 dilution; Cell Signaling Technology, Inc., Danvers, MA, USA, 9664s). The samples were then washed three times with PBS and incubated with secondary fluorescent antibodies for 1 h at 37°C in the dark to detect the primary antibodies. Nuclei were labeled with DAPI (Sigma-Aldrich, D9542) for 10 min at room temperature, and the samples were visualized under a Leica SP8 confocal fluorescence microscope (Leica Microsystems, Biberach, Germany).

### Phalloidin staining

After fixation and permeabilization, the samples were incubated with Alexa Fluor 488 or rhodamine-phalloidin (1:1,000 dilution; Invitrogen-Molecular Probes, Eugene, OR, USA) for 30 min in the dark followed by DAPI staining for 10 min for fluorescent visualization of hair cell F-actin and nuclei, respectively.

### ROS detection

Cochleae from different groups were washed in pre-warmed (37°C) PBS and stained with 5 μM MitoSox-Red (Molecular Probes, Life Technologies, 1771410) in pre-warmed PBS in the dark for 10 min. The explants were then fixed with 4% PFA.

Cellular ROS levels were detected by use of DCFH-DA (Molecular Probes, USA) and cellROX green reagent (Molecular Probes, USA). HEI-OC1 cells after treatment were washed by pre-warmed PBS and stained by 50 µM DCFH-DA or 5 μM cellROX green for 10 min or 30 min, respectively. Fluorescent images were taken with a Leica SP8 confocal fluorescence microscope.

### Mitochondrial transmembrane potential measurement

Mitochondrial membrane potential was estimated by monitoring fluorescence aggregates of JC-1 (Molecular Probes, Invitrogen, UK, T3168), TMRM (Molecular Probes, Invitrogen, UK, T668) or rhodamine 123 (Molecular Probes, Invitrogen, UK, R302). HEI-OC1 cells were treated with the designate conditions, and then incubated with 2.5 μg/ml JC-1, 20 nM TMRM, or 1 μM rhodamine 123 for 30 min at 37°C in the dark. Fluorescent images were taken with a Leica SP8 confocal fluorescence microscope.

### Caspase-mediated apoptosis assay

Cochleae or HEI-OC1 cells from different groups were washed in pre-warmed PBS and stained with 5 μM Caspase 3/7 Green Detection Reagent (Molecular Probes, Invitrogen, UK, C10723) in the dark for 10 min at 37°C. Fluorescent images were taken with a Leica SP8 confocal fluorescence microscope.

### TUNEL assay

For the TUNEL (Terminal deoxynucleotidyl transferase-mediated dUTP nick-end labeling) assay, the cochleae were rinsed with PBST three times for 10 minutes each. The cochleae were then processed using the *in situ* cell death detection Kit (Roche, Nutlet, NJ, USA; Cat. no.11684795910) according to the manufacturer's instructions.

### Protein extraction and western blot

The samples were lysed using ice-cold RIPA lysis buffer (Protein Biotechnology, PP109) with protease inhibitor cocktail (Sigma-Aldrich, 04693132001). The lysed cells were centrifuged at 12,000 × g for 10 min at 4°C. The supernatant was collected, and protein concentrations were measured using a BCA protein kit (Beyotime Institute Biotechnology, Nanjing, Jiangsu, China, P0010S). Equal amounts of each protein sample were separated by 12% SDS-PAGE and transferred onto polyvinylidene difluoride membranes (Immobilon-P, Millipore, Schaffhausen, Switzerland, IPVH00010). The membranes were blocked with 5% nonfat dried milk in Tris-buffered saline containing 0.1% Tween 20 (TBST) for 1 h at room temperature and incubated with anti-PRMT6 (1:500 dilution; Cell Signaling Technology, Inc., 14641) and anti-GAPDH overnight at 4°C. The membranes were subsequently washed three times with TBST for 10 min each and then incubated with HRP-conjugated secondary antibody (1:5,000 dilution) at room temperature for 1 h. Finally, the protein signals were visualized using the SuperSignal West Dura chemiluminescent substrate kit (Thermo Scientific, 34075). Each experiment was repeated more than three times, and all protein expression was normalized to that of GAPDH. Semi-quantification of the western blot results was performed using Image J to measure the intensities of the bands.

### ABR test

The hearing thresholds of the mice were measured by ABR analysis. The detailed methods for the ABR test were given previously 27. Briefly, mice were anesthetized (100 mg/kg ketamine and 25 mg/kg xylazine sodium, i.p.) and kept warm at 38°C with a thermostatic heating pad during the ABR recordings. The hearing threshold was assessed at five frequencies (4, 8, 16, 24, and 32 kHz) on a TDT System III apparatus (Tucker Davies Technologies, Gainesville, FL, USA).

### DPOAE test

Mice were anesthetized as described above. Response thresholds were determined provided that the 2f1 - f2 measurement was higher than two standard deviations from the noise floor. The noise floor was defined as the average of the sound levels of 10 frequency bins above and below the 2f1 - f2 frequency bin. The DPOAE responses at diction frequency 2f1-f2 were measured with two primary tones frequencies (f1 and f2, with f2/f1 = 1.2 and f2 level 10dB < f1level) to predict auditory thresholds. DPOAE response thresholds were recorded at a range of frequencies (4 kHz, 8 kHz, 16 kHz, 24 kHz and 32 kHz) within the acoustic microphone probe and the TDT system. The Level of the two primary tones will remain equal and will vary with each frequency from 80 dB SPL to 20 dB SPL in 5 dB decrements. Emissions f1 and f2 stimulated the cochlea and passed through a multifunction processor (TDT) to a computer controlled programmable attenuator, buffer amplifier, and earphone. Hearing thresholds were defined as the averaged signals for each ascertain frequency tested and the comparison with the corresponding frequency in controls.

### Cell counts

Hair cells with normal nuclei and labeled with myosin 7a were considered to be surviving hair cells. For hair cell quantification, we imaged the entire cochlea using a 40× lens on the Zeiss microscope and used Image J software to quantify the immunopositive cells. The average numbers of hair cells per 200 µm in the basal, middle, and apical turns of the cochlea were calculated from each group. For apoptotic hair cell quantification, only the middle portion of each explant (40%-60% from the apical turn) was chosen for analysis because hair cells in the apical turn are resistant to aminoglycosides. Exact numbers of cochlear explant**s** (n) are indicated in the legends.

### Statistical analysis

Student's *t*-test and ANOVA were used for comparison between two groups and among multiple groups, respectively. Statistical analyses were conducted using Microsoft Excel and GraphPad Prism 6 software. A value of *p* < 0.05 was considered statistically significant.

## Results

### PRMT6 inhibition using EPZ020411 protects mouse cochlear hair cells against neomycin- and cisplatin-induced damage

We first examined the spatial-temporal course of hair cell (HC) death in the mammalian cochlea following aminoglycoside damage. Explant cultures were exposed to neomycin at increasing concentrations (0.5 mM and 1 mM) for three different times (3 h, 6 h, and 12 h). The photomicrograph in Supplementary Fig. 1A showed three rows of outer hair cells (OHC) and a single row of inner hair cells (IHC) in a normal cochlear culture labeled with myosin 7a, a hair cell marker. We observed dose-response and time-response relationships for neomycin damage (Supplementary Figure 1). Adding 0.5 mM of neomycin to the culture medium resulted in a loss of hair cells, increasing over a time period of 3-12 h (Supplementary Figure 1B-D). Increasing the dose of neomycin to 1 mM for 6 h led to a substantial loss of hair cells (Supplementary Figure s1F), and then for 12 h resulted in a substantial loss of HCs and disrupted the normal architecture of the cochlear explants (Supplementary Figure s1G and H). This was consistent with a model in which neomycin uptake increases with its dose, and HCs more readily take up neomycin increasing over a time period. Thus, the 1 mM dose of neomycin for 6 h was used for the subsequent *in vitro* mice experiments.

**Figure 1:**
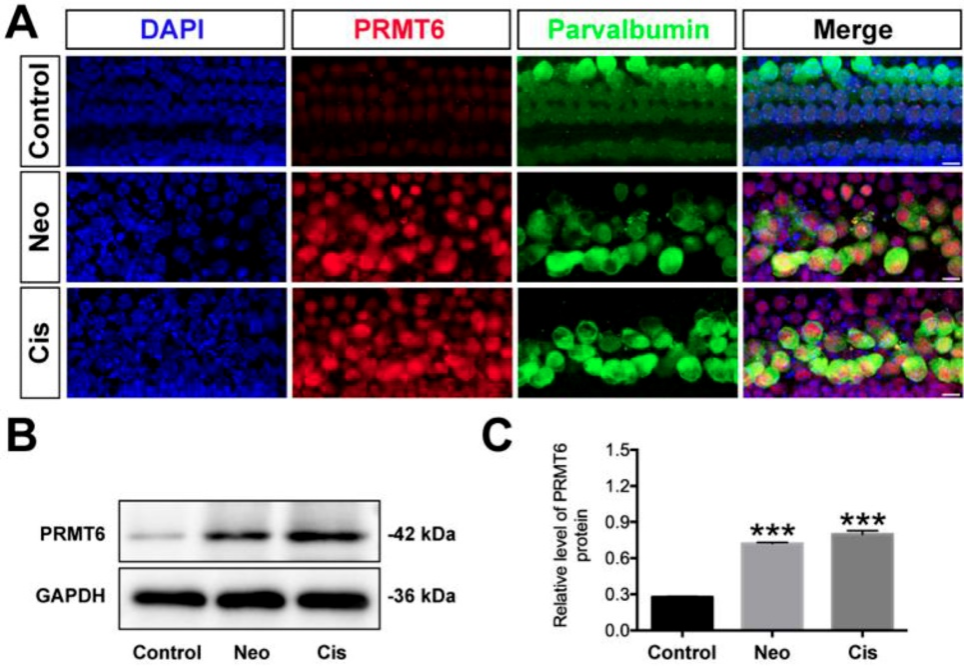
** Changes in PRMT6 induced by neomycin and cisplatin exposure.** (A) Immunofluorescence staining with PRMT6 and parvalbumin antibodies in the middle turns of the cochleae from control, neomycin (Neo), and cisplatin (Cis) groups. Scale bar = 20 μm. (B) Representative western blots showing the expression levels of PRMT6 in the cochlear explants after different treatments. (C) Semi-quantitative densitometric analyses of PRMT6 was performed using Image J. Data are presented as mean ± s.e.m. ***p < 0.001 versus the control group. n = 3 independent experiments. Neo: neomycin; Cis: cisplatin.

**Figure 2:**
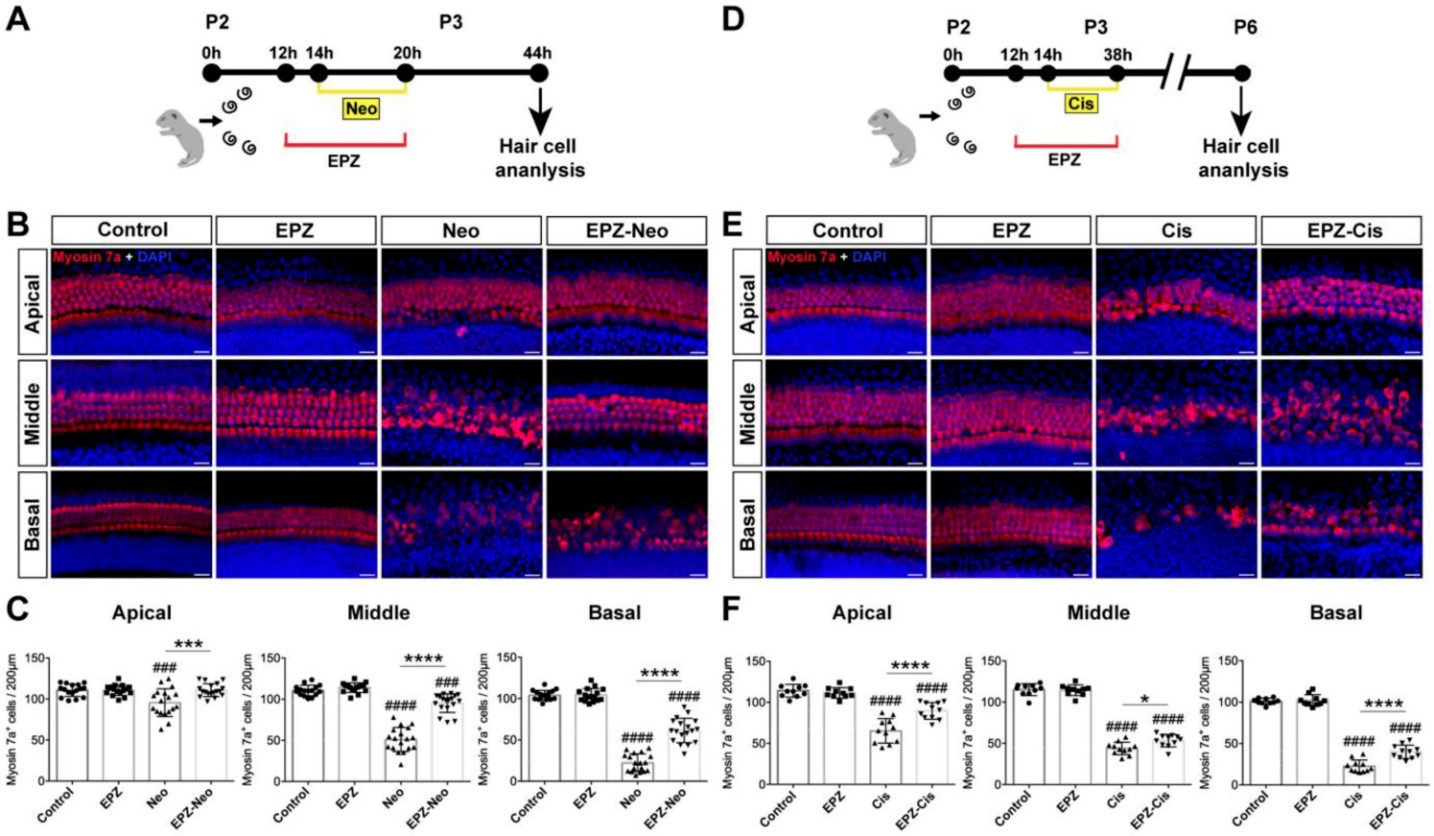
** Effects of EPZ020411 on neomycin and cisplatin-induced hair cell loss *in vitro*.** (A) Diagram of the assay for (B) and (C). (B) Immunofluorescence staining with myosin 7a (red) and DAPI (blue) in the apical, middle, and basal turns of the cochleae from control, EPZ020411-only, neomycin-only (Neo), and EPZ020411 plus neomycin (EPZ-Neo) groups. Scale bar = 20 μm. (C) Quantification of myosin 7a-positive hair cells in the apical, middle, and basal turns of the different groups. The data are expressed as the mean ± s.d. ^###^*p* < 0.001, ^####^*p* < 0.0001 versus the control group; ***p < 0.001, ****p < 0.0001 versus the neomycin group. Control group: n = 16 cochlear explants; EPZ (only EPZ020411) group: n = 16 cochlear explants; Neo (neomycin) group: n = 18 cochlear explants; EPZ-Neo (EPZ020411 + neomycin) group: n = 18 cochlear explants. (D) Experimental design for (E) and (F). (E) Immunofluorescence staining with myosin 7a (red) and DAPI (blue) in the apical, middle, and basal turns of the cochleae from control, EPZ020411-only, cisplatin-only (Cis) and EPZ020411 plus cisplatin (EPZ-Cis) groups. Scale bar = 20 μm. (F) Hair cells positive for myosin 7a fluorescence were counted every 200 μm along the apical, middle, and basal regions of the cochlear explants from different groups. The data are presented as the mean ± s.d. ^####^*p* < 0.0001 versus the control group; *p < 0.05, ****p < 0.0001 versus the cisplatin group. Control group: n = 10 cochlear explants; EPZ (only EPZ020411) group: n = 10 cochlear explants; Cis (cisplatin) group: n = 11 cochlear explants; EPZ-Cis (EPZ020411 + cisplatin) group: n = 11 cochlear explants.

To examine whether PRMT6 expression level in cochlear HCs was affected by ototoxic drugs, we treated the cultured cochleae with neomycin and cisplatin. As shown in Fig. 1, the level of PRMT6 significantly increased in the cochlear explants after neomycin and cisplatin treatment compared to undamaged controls (Figure 1). These results demonstrated that neomycin and cisplatin led to increase in the expression of PRMT6 in HCs within 6 h and 24 h, respectively, indicating that PRMT6 might be involved in the process of ototoxic drugs-induced HC injury. In order to investigate the effect of PRMT6 on neomycin‐induced HC damage, cochlear explants were treated with EPZ020411, a small molecule PRMT6 inhibitor. The cochlear explants were divided into following groups: undamaged control group; neomycin-only treatment for 6 h; 2 h (20 μM, 40 μM) EPZ020411 pre-treatment before neomycin treatment for 6 h; co-treatment of (20 μM, 40 μM) EPZ020411 and neomycin for 6 h; and 24 h (20 μM, 40 μM) EPZ020411 post-treatment after neomycin for 6 h (Supplementary Figure 2A). The survivals of HCs across middle segments of the cochlear explants were detailed in Supplementary Figure 2. A dose-dependent protection on cochlear hair cells by EPZ020411 against neomycin ototoxicity was observed and more surviving HCs were found in the pre-treatment with 40 μM EPZ020411 group than the other groups in the middle segments (Supplementary Figure 2). The number of surviving hair cells in the 40 μM EPZ020411 pre-treatment group was also significantly higher than in the neomycin only controls, and there was no significant change in HC damage between the post-treatment group and neomycin-only controls (Supplementary Figure 2). Therefore, we chose a concentration of 40 μM EPZ020411 pretreatment for the subsequent experiments. Furthermore, we quantified the number of HCs by myosin 7a staining on each turn of the base, middle, and apical parts after each set of treatment in the cultured cochlear explants. As shown in Fig. 2, after 24 h recovery, cultured in normal medium or with 40 μM EPZ020411-only for 6 h, the HC numbers appeared no changes, indicating that addition of EPZ020411 itself to the cochlear explant cultures did not induce any apparent morphological changes (Figure 2A and 2B). After neomycin challenge, HC loss was most obvious in the basal and middle turns, while apical HCs remained largely intact and no significant difference was seen compared to the undamaged controls (Figure 2B). In contrast, EPZ020411 treatment prior to neomycin challenge showed significantly more HC survival in comparison with the neomycin-only group (Figure 2B and 2C), revealing a protective effect by EPZ020411.

We next investigated whether EPZ020411 protects HC against cisplatin-induced damage in cultured cochlear explants from P2 mice (Figure 2D). Cochlea explants treated with 20 μM cisplatin for different times (12 h, 24 h, and 48 h), and cisplatin treatment for 24 h showed extensive degeneration of both IHCs and OHCs in the basal and middle turns of the cochlea (Supplementary Figure s3), thus the cisplatin treatment for 24 h was chose as an appropriate condition for HC injury. In contrast, pretreatment with 40 μM EPZ020411 exhibited protective effects against the cisplatin induced loss of HCs. Cochlear explants treated with EPZ020411 alone did not exhibit any damage to the HCs (Figure 2E). Furthermore, we quantified the number of HCs on each turn of the base, middle, and apical parts after each set of treatment in the cultured cochlear explants. As shown in Fig. 2F, there were a mean of 22.1 ± 2.9, 45.6 ± 2.9, and 64.7 ± 5.6 hair cells per 200 µm in the basal, middle, and apical turns, respectively, of the cochlear explants treated with cisplatin alone, while pretreatment with EPZ020411 resulted in a mean of 36.5 ± 2.6, 57.9 ± 3, and 89.8 ± 3.8 hair cells per 200 µm in the basal, middle, and apical turns of the cochlear explants (Figure 2E and 2F). Taken together, these results clearly indicated that EPZ020411 protects HCs from cisplatin-induced cell death.

### EPZ020411 ameliorates neomycin- and cisplatin-induced apoptosis and attenuates the activation of the mitochondrial apoptotic pathway

**Figure 3:**
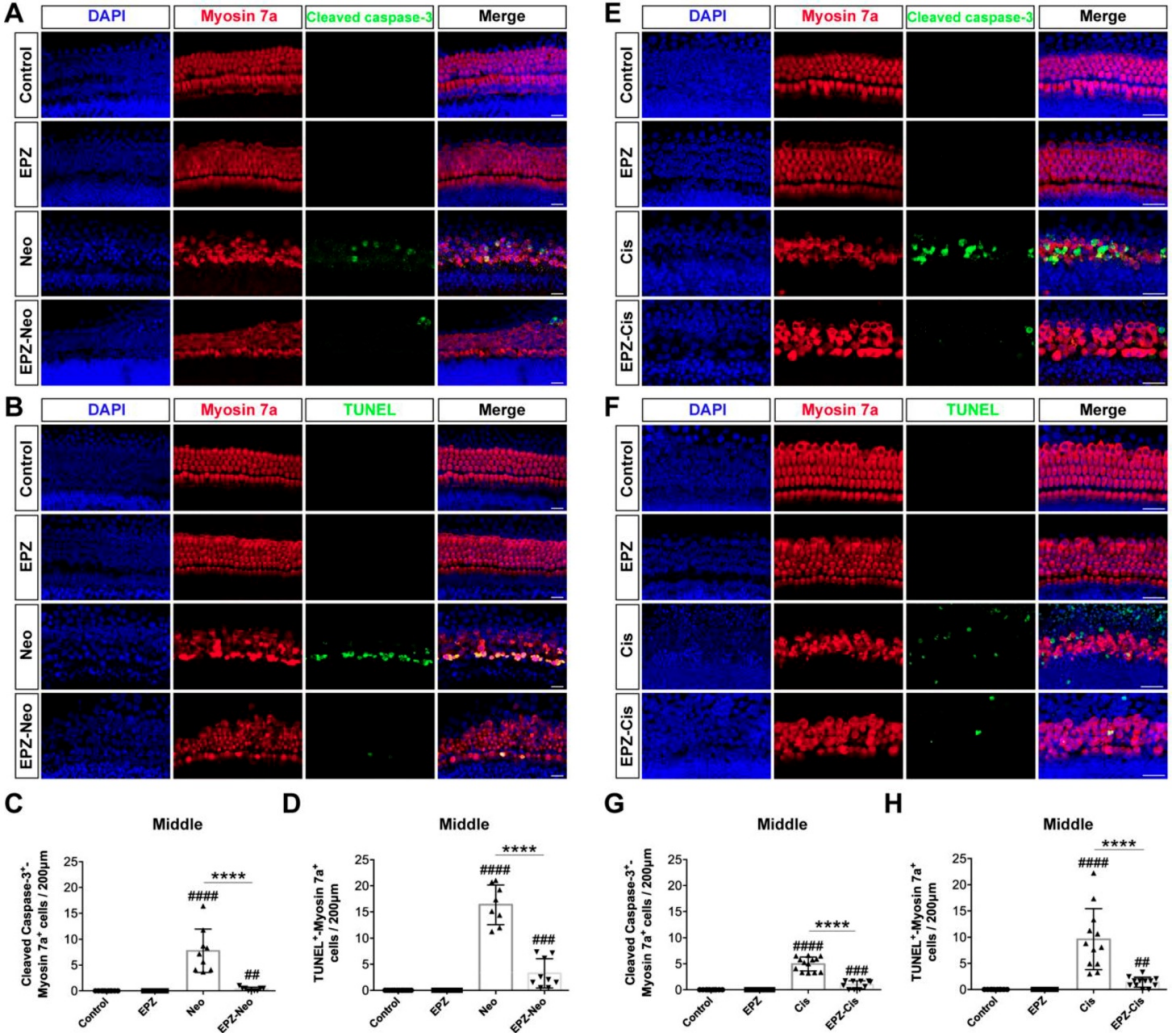
** Effects of EPZ020411 on cell apoptosis in the mouse cochlea after neomycin and cisplatin injury.** (A) Immunofluorescence staining with cleaved caspase-3 (green) and myosin 7a (red) in the middle turns of the cochleae from the control, EPZ020411, neomycin (Neo), and EPZ020411 plus neomycin (EPZ-Neo) groups. (B) Immunofluorescence staining for TUNEL (green) and myosin 7a (red) in the middle turns of the cochleae from each treatment group. Scale bars = 20 μm. (C) Quantification of the number of cleaved caspase-3/myosin 7a double-positive cells in the middle turns of the cochleae after the different treatments. The data are presented as the mean ± s.d. ^##^*p* < 0.01,^ ####^*p* < 0.0001 versus the control group; ****p < 0.0001 versus the neomycin group. Control group: n = 10 cochlear explants; EPZ (only EPZ020411) group: n = 10 cochlear explants; Neo (neomycin) group: n = 9 cochlear explants; EPZ-Neo (EPZ020411 + neomycin) group: n = 9 cochlear explants. (D) Quantification of the number of TUNEL/myosin 7a double-positive cells in the middle turns of the cochleae after the different treatments. The data are presented as the mean ± s.d. ^###^*p* < 0.001,^ ####^*p* < 0.0001 versus the control group; ****p < 0.0001 versus the neomycin group. Control group: n = 14 cochlear explants; EPZ (only EPZ020411) group: n = 14 cochlear explants; Neo (neomycin) group: n = 8 cochlear explants; EPZ-Neo (EPZ020411 + neomycin) group: n = 9 cochlear explants. (E and F) Immunofluorescence staining with myosin 7a and cleaved caspase-3 (E) or TUNEL (F) in the middle turns of the cochleae from different groups. (G) Quantification of the number of Cleaved caspase-3/myosin 7a double-positive cells in the middle turns of the cochleae after the different treatments. Data are shown as the mean ± s.d. ^###^*p* < 0.001,^ ####^*p* < 0.0001 versus the control group; ****p < 0.0001 versus the cisplatin group. Control group: n = 10 cochlear explants; EPZ (only EPZ020411) group: n = 18 cochlear explants; Cis (cisplatin) group: n = 13 cochlear explants; EPZ-Cis (EPZ020411 + cisplatin) group: n = 10 cochlear explants. (H) Quantification of the number of TUNEL/myosin 7a double-positive cells in the middle turns of the cochleae after the different treatments. Data are shown as the mean ± s.d. ^##^*p* < 0.01, ^####^*p* < 0.0001 versus the control group; ****p < 0.0001 versus the cisplatin group. Control group: n = 10 cochlear explants; EPZ (only EPZ020411) group: n = 10 cochlear explants; Cis (cisplatin) group: n = 12 cochlear explants; EPZ-Cis (EPZ020411 + cisplatin) group: n = 12 cochlear explants.

To investigate whether PRMT6 inhibition could reduce cell apoptosis in cultured cochleae, we analyzed apoptosis by cleaved caspase-3 and TUNEL staining. The results showed that number of cleaved caspase-3/myosin 7a double-positive cells was significantly increased in the neomycin group compared to the control group (Figure 3A and 3C). Increased hair cell apoptosis was further confirmed by a similar increase in the number of TUNEL/myosin 7a double-positive cells in the neomycin group (Figure 3B and 3D). The EPZ020411 pretreated group showed dramatically decreased numbers of cleaved caspase-3/myosin 7a double-positive and TUNEL/myosin 7a double-positive cells compared with the neomycin-only group (Figure 3C and D). These results indicated that pretreatment with EPZ020411 might suppress the apoptotic cascade induced by aminoglycosides. We also determined the nature of the anti-apoptotic roles of EPZ020411 in cisplatin-damaged cochlear explants by assessing the cleaved caspase-3 and myosin 7a staining (Figure 3E). Immunohistochemistry analysis showed that there was significant hair cell loss after cisplatin treatment as indicated by increased cleaved caspase-3 immunoreactivity and that EPZ020411 significantly attenuated this hair cell loss (Figure 3G). Hair cell death was also verified by staining for DNA fragmentation with the TUNEL assay (Figure 3F). More TUNEL-positive apoptotic hair cells were also observed in explants treated with cisplatin than in explants pre-treated with EPZ020411 (Figure 3H). These results demonstrated that EPZ020411 prevented cisplatin-induced apoptosis in the hair cells of the cochlear explants.

### EPZ020411 decreases ROS generation in neomycin- and cisplatin-damaged cochlear explants

**Figure 4:**
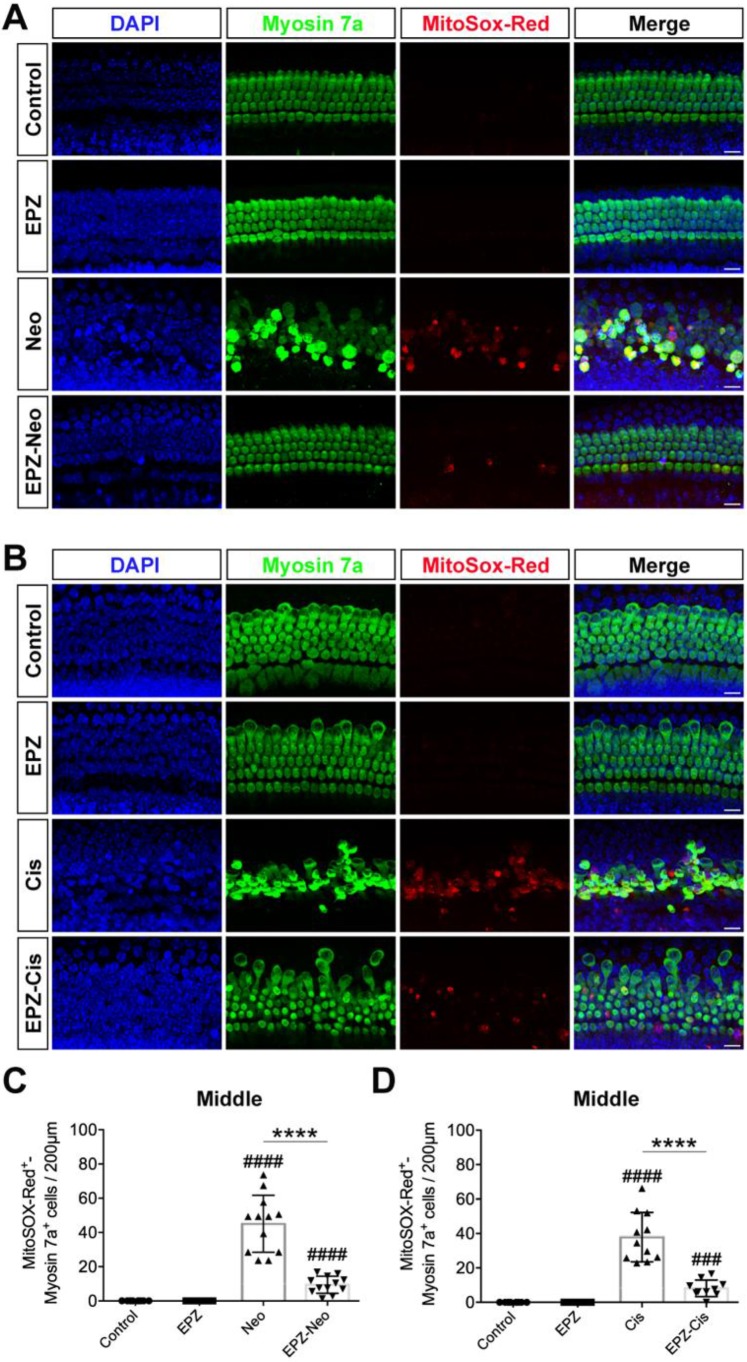
** Effects of EPZ020411 on ROS production in neomycin and cisplatin-damaged cochlear explants.** (A-B) Immunofluorescence staining with MitoSox-Red (red) and myosin 7a (green) in the middle turns of the cochleae from different groups. Scale bars = 20 μm. (C) Quantification of MitoSox-Red/myosin 7a double-positive cells in the middle turns of the different groups. The data are expressed as the mean ± s.d. ^####^*p* < 0.0001 versus the control group; ****p < 0.0001 versus the neomycin group. Control group: n = 8 cochlear explants; EPZ (only EPZ020411) group: n = 8 cochlear explants; Neo (neomycin) group: n = 12 cochlear explants; EPZ-Neo (EPZ020411 + neomycin) group: n = 12 cochlear explants. (D) Quantification of MitoSox-Red/myosin 7a double-positive cells in the middle turns of the different groups. The data are expressed as the mean ± s.d. ^###^*p* < 0.001,^ ####^*p* < 0.0001 versus the control group; ****p < 0.0001 versus the cisplatin group. Control group: n = 8 cochlear explants; EPZ (only EPZ020411) group: n = 8 cochlear explants; Cis (cisplatin) group: n = 11 cochlear explants; EPZ-Cis (EPZ020411 + cisplatin) group: n = 10 cochlear explants.

It has been reported by several groups that the accumulation of ROS plays an important role in the process of hair cell death by activating multiple apoptotic pathways 29. MitoSox-Red staining was used to monitor mitochondrial ROS production in cochlear explants after the different treatments. As shown in Fig. 4A, there were almost no MitoSox-Red-positive cells in the untreated control group or the EPZ020411-only group. ROS production was significantly increased after treatment with neomycin for 6 h compared to undamaged controls, and this increase was significantly inhibited by EPZ020411 pretreatment (Figure 4A and C). The generation of ROS has also long been recognized as an important contributor to cisplatin-induced apoptosis. We therefore used MitoSox-Red to determine whether EPZ020411 could reduce the mitochondrial ROS generated from cisplatin exposure in cochlear explants (Figure 4B). In the EPZ020411 plus cisplatin group, a significant decrease in red fluorescence was observed and MitoSox-Red/myosin 7a double-positive cells were greatly decreased compared to the cisplatin only group (Figure 4D).

### EPZ020411 protects against neomycin-induced hearing loss *in vivo*

**Figure 5:**
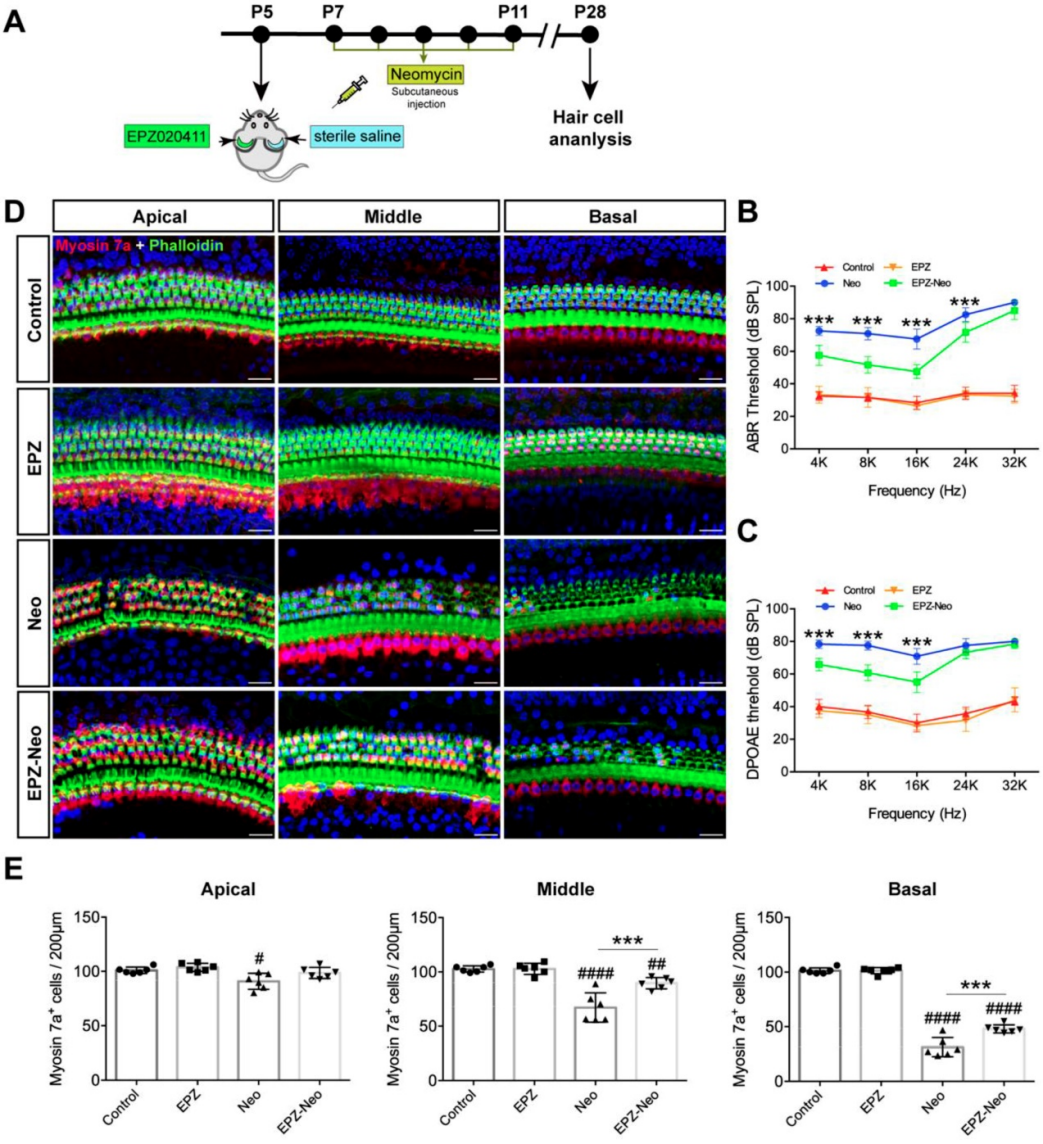
** Effects of EPZ020411 on neomycin-induced chronic ototoxicity *in vivo*.** (A) Experimental workflow for (B-E). (B-C) ABR and DPOAE thresholds were analyzed in mice treated with or without a combination of EPZ020411/sterile saline and neomycin. The data are expressed as the mean ± s.d. ***p < 0.001. EPZ (only EPZ020411) group: n = 6 left ears; Control group (only sterile saline): n = 6 right ears; EPZ-Neo (EPZ020411 + neomycin) group: n = 6 left ears; Neo (sterile saline + neomycin) group: n = 6 right ears; Left and right ears of EPZ and Control groups from the same mice, and left and right ears of EPZ-Neo and Neo groups from the same mice. (D) Immunofluorescence staining with myosin 7a (red) and phalloidin (green) in the apical, middle, and basal turns of the cochleae from different groups. Scale bars = 20 μm. (E) Quantification of myosin 7a-positive hair cells in the apical, middle, and basal turns of the cochleae from different groups. The data are expressed as the mean ± s.d. ^#^*p* < 0.05, ^##^*p* < 0.01, ^####^*p* < 0.0001 versus the control group; **p < 0.01 and ***p < 0.001 versus the neomycin group. EPZ (only EPZ020411) group: n = 6 left ears; Control group (only sterile saline): n = 6 right ears; EPZ-Neo (EPZ020411 + neomycin) group: n = 6 left ears; Neo (sterile saline + neomycin) group: n = 6 right ears; Left and right ears of EPZ and Control groups from the same mice, and left and right ears of EPZ-Neo and Neo groups from the same mice.

To evaluate the protective potential of EPZ020411 on aminoglycoside-induced chronic models of ototoxicity *in vivo*, we injected C57BL/6J wild-type mice at P5 with 10 mM EPZ020411 for 1μl in the left ear and the same volume of sterile saline in the right ear. After 2 days treatment, the mice received systemic administration of 20 mg/ml neomycin for 0.01 ml/g by subcutaneous injection for five consecutive days (indicated as Neo and EPZ-Neo) (Figure 5A). In addition, only EPZ020411-pretreated or sterile saline-pretreated mice that did not receive neomycin administration were used as negative controls, and indicated as EPZ020411 and control, respectively, in the Fig. 5B-D. To determine whether EPZ020411 preserves hearing function, the hearing function of mice were determined by ABR and DPOAE measurements. As shown in Fig. 5B and C, after neomycin administration, the sterile saline-treated ears had significantly elevated thresholds at all tested frequencies, whereas the EPZ020411-injected ears exhibited significantly reduced thresholds elevation. The cochleae were harvested immediately after testing and dissected into the apical, middle, and basal turns, and the numbers of surviving hair cells labeled with myosin 7a in the three cochlear turns were imaged and quantified (Figure 5D and 5E).

Fig. 5D showed that no HC loss was observed in EPZ020411 only and sterile saline only control groups, but neomycin treatment induced extensive degeneration of hair cells in the middle and basal turns of the cochlea. However, the numbers of hair cells were significantly increased in the EPZ020411-pretreated ears compared to the contralateral neomycin only damaged ears (Figure 5E). To visualize the hair bundles, stereocilia containing F-actin were labeled with phalloidin. After neomycin exposure, hair bundles were primarily observed in the apical segment. The outer hair cell bundles were missing in middle and basal segments, whereas the cochleae pretreated with EPZ020411 had more surviving outer hair cell bundles in the basal and middle turns in comparison with the contralateral cochleae (Figure 5D).

**Figure 6:**
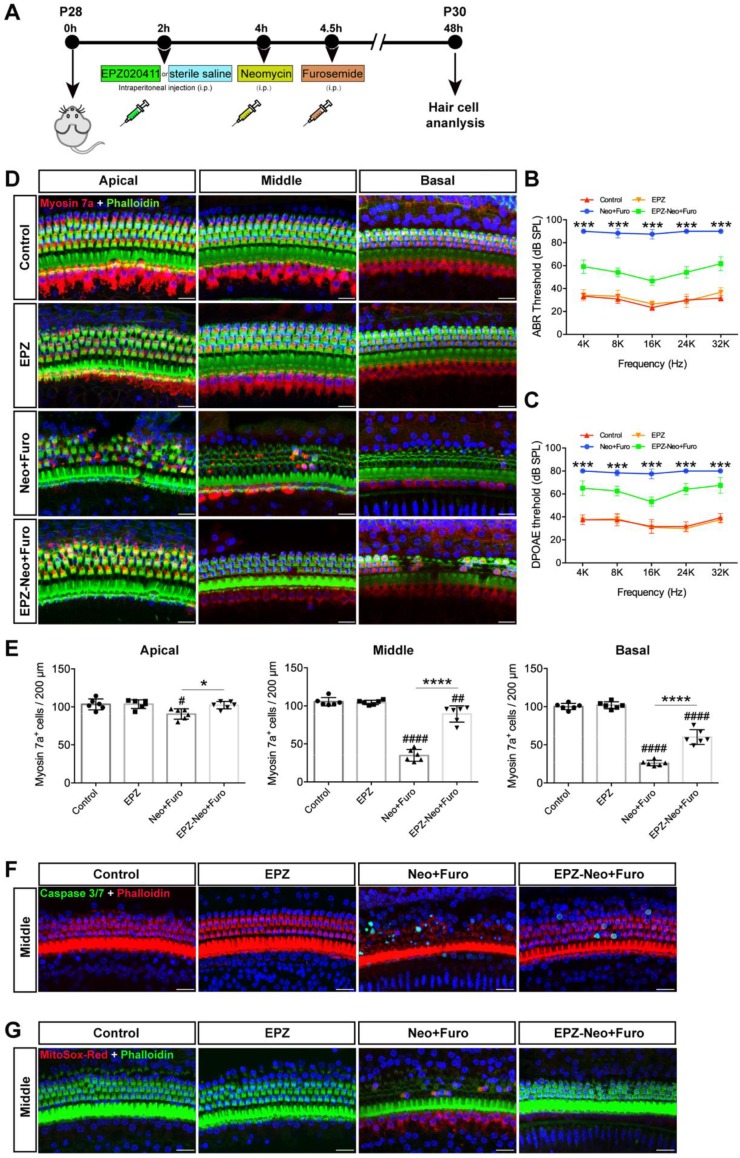
** Effects of EPZ020411 on neomycin-induced acute hearing loss *in vivo*.** (A) The experimental workflow for (B-G). (B-C) ABR and DPOAE thresholds were analyzed in mice treated with a combination of EPZ020411/sterile saline and neomycin + furosemide. The data are expressed as the mean ± s.d. ***p < 0.001. n = 6 left ears from 6 mice per group. (D) Representative images of hair cells staining for labeled with myosin 7a (red) and phalloidin (green) in the apical, middle and basal turns of different groups. Scale bars = 20 μm. (E) Quantification of myosin 7a-positive hair cells in the apical, middle and basal turns of different groups. The data are expressed as the mean ± s.d. ^#^*p* < 0.05, ^##^*p* < 0.01, ^####^*p* < 0.0001 versus the control group; *p < 0.05 and ****p < 0.0001 versus the neomycin + furosemide group. n = 6 left ears from 6 mice per group. (F-G) Immunofluorescence staining with Caspase 3/7 (green) and phalloidin (red) (F) or MitoSox-Red (red) and phalloidin (green) (G) in the middle turns of the cochleae from different groups. Scale bars = 20 μm. EPZ: only EPZ020411; Control: only sterile saline; Neo+Furo: sterile saline + neomycin + furosemide; EPZ-Neo+Furo: EPZ020411 + neomycin + furosemide; i.p.: intraperitoneal.

Next, to assess the protective potential of EPZ020411* in vivo* in an acute ototoxicity model, we used neomycin in conjunction with furosemide, a loop diuretic known to facilitate neomycin crossing the blood-labyrinth barrier, to cause acute ototoxicity. We injected both sexes of C57BL/6J wild-type mice at P28 with 10 mM EPZ020411 for 10 mg/kg 2 h before neomycin treatment. After pretreatment, the mice received systemic administration of neomycin (100 mg/kg) in conjunction with intraperitoneal injection of furosemide (300 mg/kg) (Figure 6A). EPZ020411-pretreated or sterile saline-pretreated mice that did not receive neomycin and furosemide administration were used as controls, and indicated as EPZ020411 and control, respectively, in the Fig. 6. As shown in Fig. 6B and C, after neomycin + furosemide administration, the control ears had significantly elevated ABR and DPOAE thresholds, whereas the EPZ020411-injected ears exhibited significantly reduced thresholds elevation. Then the cochleae were harvested, and the surviving hair cells and hair bundles were labeled with myosin 7a and phalloidin, respectively (Figure 6D). Fig. 6E showed that neomycin with furosemide caused extensive loss of hair cells in a base-to-apex gradient; and pretreatment with EPZ020411 significantly reduced neomycin + furosemide-induced HC loss. Note that mice received furosemide alone (no neomycin) combined with EPZ020411 or sterile saline had no effect (Supplementary Figure s4).

To determine the cell apoptosis in the HCs under the different treatment conditions, phalloidin and caspase 3/7 were both applied to reveal the apoptotic signals (Figure 6F). There was no distribution of caspase 3/7 within the HCs in the control group. Neomycin + furosemide administration induced more caspase 3/7-positive cells accompanied by the reduction of HC numbers (26.57 ± 7.583 per 200 μm in the middle turn; n = 6 ears, *p* < 0.0001), whereas treatment of EPZ020411 could significantly reduce caspase 3/7-positive cells induced by acute ototoxic insult and conserved more HCs (4.037 ± 3.254 per 200 μm in the middle turn; n = 6 ears,* p* < 0.0001 vs. neomycin + furosemide; Figure 6F). Because EPZ020411 is shown to decrease ROS production from the whole epithelium of the cochlea mediated by neomycin *in vitro*, we next performed* in vivo* experiments to determine whether ROS production could be attenuated by EPZ020411 administration (Figure 6G). In the control group, no MitoSox-Red was distributed in the HC cells. After 2 days of neomycin + furosemide treatment, more MitoSox-Red-positive cells were detected (37.49 ± 8.174 per 200 μm in the middle turn; n = 6 ears) compared with the controls (*p* < 0.0001). In contrast, EPZ020411 significantly reduced the ROS production triggered by neomycin + furosemide (1.513 ± 1.747 per 200 μm in the middle turn; n = 6 ears,* p* < 0.0001 vs. neomycin + furosemide; Figure 6G).

### EPZ020411 protects against cisplatin-induced hearing loss *in vivo*

**Figure 7:**
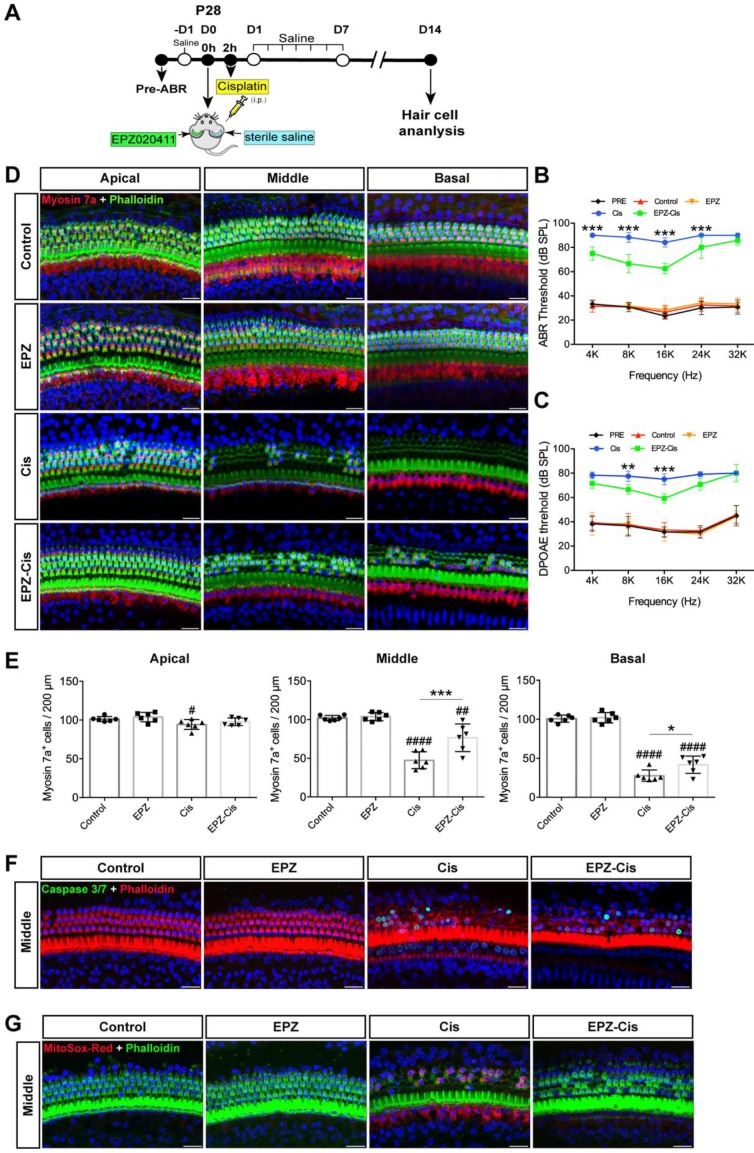
** Effects of EPZ020411 on cisplatin-induced hearing loss *in vivo*.** (A) Experimental design for (B-G). (B-C) ABR and DPOAE thresholds were analyzed in mice treated with a combination of EPZ020411/sterile saline and cisplatin. The data are expressed as the mean ± s.d. **p < 0.01 and ***p < 0.001. EPZ (only EPZ020411) group: n = 6 left ears; Control group (only sterile saline): n = 6 right ears; EPZ-Cis (EPZ020411 + cisplatin) group: n = 6 left ears; Cis (sterile saline + cisplatin) group: n = 6 right ears; Left and right ears of EPZ and Control groups from the same mice, and left and right ears of EPZ-Cis and Cis groups from the same mice. (D) Immunofluorescence images of the apical, middle, and basal turns of the cochleae double stained with phalloidin and myosin 7a. Scale bars = 20 μm. (E) Comparison of the percentage of myosin 7a-positive hair cell in all three regions of the different groups. Data are shown as the mean ± s.d. ^#^*p* < 0.05, ^##^*p* < 0.01, ^####^*p* < 0.0001 versus the control group; *p < 0.05 and **p < 0.01 versus the cisplatin group. EPZ (only EPZ020411) group: n = 6 left ears; Control group (only sterile saline): n = 6 right ears; EPZ-Cis (EPZ020411 + cisplatin) group: n = 6 left ears; Cis (sterile saline + cisplatin) group: n = 6 right ears; Left and right ears of EPZ and Control groups from the same mice, and left and right ears of EPZ-Cis and Cis groups from the same mice. (F-G) Immunofluorescence staining with Caspase 3/7 (green) and phalloidin (red) (F) or MitoSox-Red (red) and phalloidin (green) (G) in the middle turns of the cochleae from different groups. Scale bars = 20 μm. i.p.: intraperitoneal.

To examine whether EPZ020411 protects against cisplatin-induced hearing loss* in vivo*, we injected both sexes of C57BL/6J wild-type mice at P28 trans-tympanically with EPZ020411 in the left ear and sterile saline in the right ear, and 2 h later we injected 30 mg/kg cisplatin (i.p.) to affect both ears (indicated as Cis and EPZ-Cis) (Figure 7A). This same procedure has been used previously in setting up animal models of hearing loss 28. In addition, only EPZ020411-pretreated or sterile saline-pretreated mice that did not receive cisplatin administration were used as negative controls, and indicated as EPZ020411 and control, respectively, in the Fig. 7. We first established the cisplatin time responses for hearing loss in mice. The ABR thresholds were measured at 2, 4, 7 and 14 d (D2, D4, D7 and D14, respectively) after drug exposure (sterile saline + cisplatin or EPZ020411 + cisplatin). At D 2, sterile saline-pretreated control ears showed slight ABR threshold elevation at 16 kHz and 32 kHz, whereas EPZ020411 pretreatment produced no change in ABR thresholds when compared to Pretreatment thresholds; and there was no loss of hair cells in both sterile saline + cisplatin and EPZ020411 + cisplatin ears (Supplementary Figure 5E and 5F). At D4 and D7, sterile saline-pretreated ears showed evident ABR threshold elevation at all tested frequencies, whereas EPZ020411-pretreated ears exhibited a reduced ABR threshold elevation. Morphological analysis showed that there was a loss of HCs at basal and middle turns in sterile saline-pretreated control ears but a significant reduced loss of HCs in EPZ020411-pretreated ears (Supplementary Figure 5E and 5F). ABR and DPOAE measurements 14 days after exposure demonstrated a significant elevation in hearing thresholds at all test frequencies, whereas pretreatment with EPZ020411 diminished thresholds elevation (Figure 7B and 7C). Immunofluorescence staining showed that cisplatin-induced hearing loss was associated with a reduction in HC number, assessed at the basal and middle turns (Figure 7D and 7E). This loss of HCs was significantly reduced by EPZ020411 pretreatment (Figure 7D and 7E). Additionally, more caspase 3/7-positive cells were observed in ears treated with sterile saline + cisplatin (22.01 ± 4.435 per 200 μm in the middle turn; n = 6 ears) than in ears pre-treated with EPZ020411 + cisplatin (4.018 ± 3.684 per 200 μm in the middle turn; n = 6 ears, *p* < 0.0001; Figure 7F). A similar change was observed at the MitoSox-Red-positive cells (sterile saline + cisplatin: 43.62 ± 6.740 vs EPZ020411 + cisplatin: 0.9616 ± 0.9760 per 200 μm in the middle turn; n = 6 ears per group, *p* < 0.0001; Figure 7G). Taken together, these data suggested that EPZ020411 indeed attenuated oxidative DNA damage and reduced subsequent apoptosis-mediated hair cell loss after cisplatin exposure, at least partly accounting for its protective role against cisplatin-induced hearing loss.

### Downregulation of PRMT6 decreases cell apoptosis and ROS production in HEI-OC1 cells after cisplatin exposure

The House Ear Institute-Organ of Corti 1 (HEI-OC1) cell line is a widely used progenitor hair cell line derived from the auditory organ of mouse 30. This cell line is sensitive to ototoxins including aminoglycosides and cisplatin and was developed as an* in vitro* system for screening for protective compounds and for investigating the molecular and cellular mechanisms involved in ototoxicity. In order to investigate the role of PRMT6 in ototoxic drugs-induced cell death in the HEI-OC1 cell line, we knocked down PRMT6 by siRNA. We designed three PRMT6-siRNA constructs (siRNA-01, siRNA-02, siRNA-03) and used them to transfect the HEI-OC-1 cell line, and the siRNA control group was HEI-OC1 cells transfected with negative siRNA (Negative-siRNA).

qRT-PCR results showed that mRNA levels of PRMT6 was significantly reduced after transfection with siRNA-01, siRNA-02, and siRNA-03. The lowest PRMT6 expression was detected when HEI-OC-1 cells were transfected with siRNA-03 (Supplementary Figure 6A). To further confirm our findings, we performed western blot analysis and found significantly decreased expression of PRMT6 protein after transfection with the siRNA-03 (Supplementary Figure 6B and 6C). Moreover, the immunofluorescence staining also showed that the expression of PRMT6 in HEI-OC-1 cells was significantly downregulated after transfection with the siRNA-03 (Supplementary Figure 6D). These results suggested that negative-siRNA transfection has no effect on PRMT6 expression, while PRMT6 siRNA-03 transfection effectively inhibits PRMT6 expression in HEI-OC1 cells, thus we used the PRMT6 siRNA-03 in the following experiments and indicated as PRMT6-siRNA.

We first investigated whether downregulation of PRMT6 alone leads to cell apoptosis without cisplatin damage. We quantified the cell apoptosis by caspase3/7 staining. Our results showed that knockdown of PRMT6 alone do not cause significant apoptosis in HEI-OC-1 cells without cisplatin treatment (Supplementary Figure s6E). Next, we inhibited PRMT6 with siRNA to investigate the role of PRMT6 in the cisplatin-induced cell death of HEI-OC-1 cells. As previous studies have shown that treatment of HEI-OC1 cells with 30 μM cisplatin can effectively induce cell apoptosis at 24 h 31, we selected 30 μM as the treatment concentration in the following experiments. HEI-OC1 cells were divided into the following groups: control HEI-OC-1 cells without any treatment (control), the cells transfected with negative siRNA and cisplatin (Cis Negative-siRNA), the HEI-OC-1 cells treated with cisplatin only (Cis), and the PRMT6-siRNA-transfected HEI-OC-1 cells treated with cisplatin (Cis PRMT6-siRNA). After 24 h of cisplatin treatment, the cells were stained with caspase 3/7 to evaluate apoptotic cells. We found significantly more caspase 3/7-positive cells in the cisplatin-treated group compared with the undamaged control cells. In contrast, the PRMT6-siRNA-transfected cells had significantly fewer caspase 3/7-positive cells compared with the controls transfected with negative siRNA (Supplementary Figure 6E and 6F). Taken together, these results demonstrated that downregulation of PRMT6 significantly reduces the apoptosis of HEI-OC-1 cells after cisplatin damage, indicating that downregulation of PRMT6 attenuates the sensitivity of HEI-OC-1 cells to cisplatin damage.

**Figure 8:**
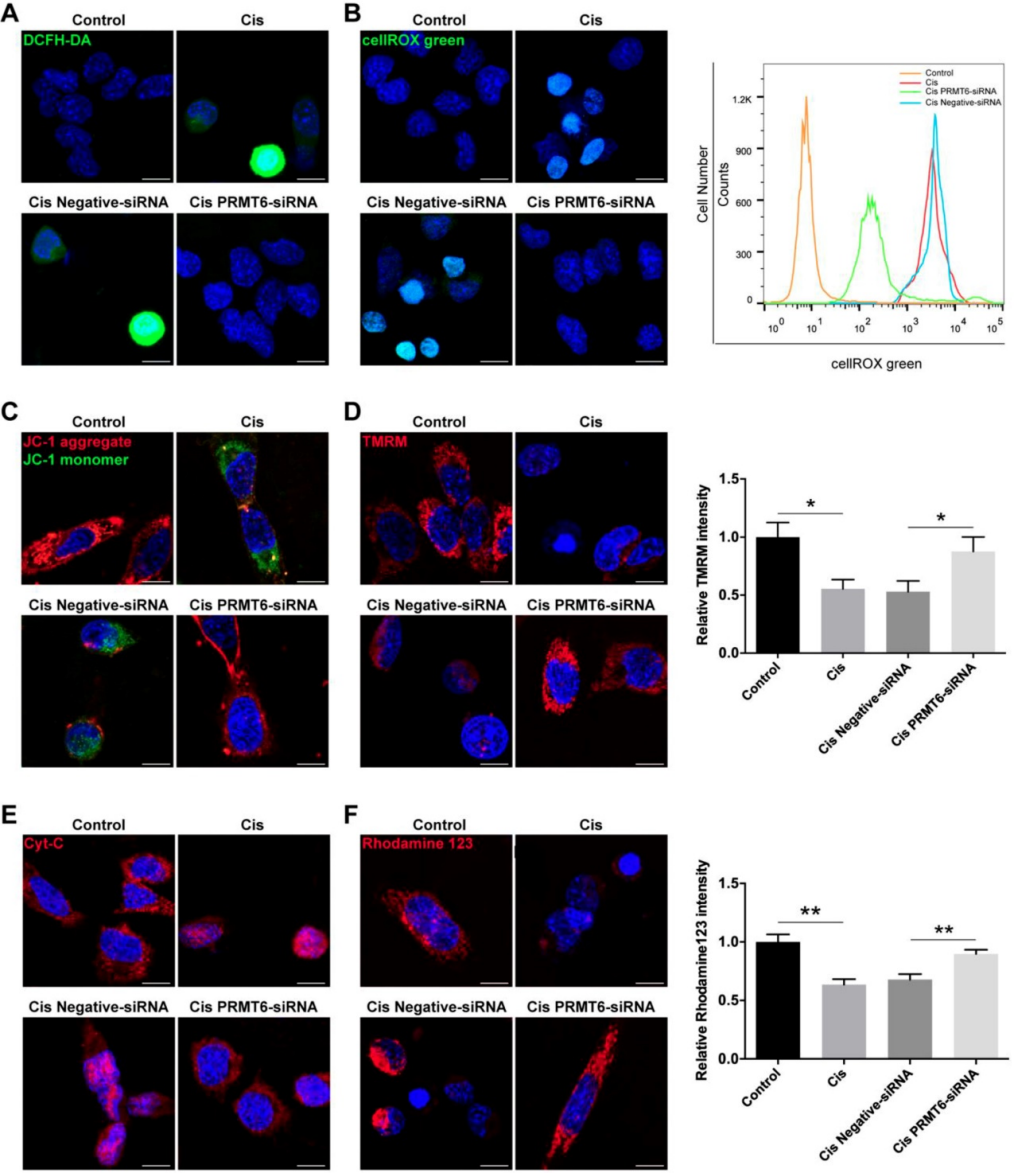
** Effects of PRMT6 knockdown on** cisplatin**-induced mitochondrial apoptosis in** HEI-OC1 **cells.** (A-B) Measurement of intracellular reactive oxygen species (ROS) production. Representative microscopic appearance of HEI-OC1 cells stained with DCFH-DA (A) and cellROX green (B). Quantitative changes in the cellROX green was analyzed by flow cytometry data. (C and D) Representative fluorescence images of HEI-OC1 cells stained with JC-1 (C) and TMRM (D). Quantitative changes in the TMRM fluorescence intensity was analyzed by Image J. Data represent the mean ± s.e.m. *p < 0.05. (E) Immunostaining of Cyt-c was detected by fluorescence microscopy. (F) Representative fluorescence images of HEI-OC1 cells stained with rhodamine 123. Quantitative changes in the rhodamine 123 fluorescence intensity was analyzed by Image J. Data represent the mean ± s.e.m. **p < 0.01. Scale bars = 10 µm. Cis: cisplatin.

Previous studies have shown that treatment of HEI-OC1 cells with cisplatin induces apoptosis through the production of ROS 32. Therefore, we determined whether PRMT6 knockdown regulates intracellular levels of ROS in cisplatin-damaged HEI-OC1 cells. Following treatment with cisplatin, intracellular levels of ROS were determined by using DCFH-DA (Figure 8A). Immunohistochemistry result showed that DCFH-DA intensity was significantly increased after cisplatin treatment compared with the undamaged controls, which indicates that these HEI-OC1 cells produced a significant amount of ROS. In contrast, DCFH-DA intensity was significantly decreased in the PRMT6-siRNA-transfected cells compared with the negative siRNA controls (Figure 8A). Accumulation of ROS was also confirmed by using the ROS indicator dye cellROX green, which labels a series of intracellular compartments, including cytoplasm, nucleus, and mitochondria. Both immunohistochemistry and flow cytometry results showed that the ROS levels were increased after cisplatin treatment compared to the undamaged controls (Figure 8B), and downregulation of PRMT6 by siRNA transfection significantly decreased the ROS levels in HEI-OC-1 cells compared with the negative siRNA controls (Figure 8B). These results suggested that PRMT6 knockdown significantly attenuates intracellular ROS levels in HEI-OC1 cells after cisplatin injury.

Ototoxic drugs can trigger intrinsic apoptosis in auditory sensory HCs, and the mitochondria are the main conductors of the intrinsic apoptotic pathway. The first event in the dysregulation of mitochondrial dynamics is the loss of mitochondrial membrane potential (ΔΨm), an important indicator of mitochondrial function, consequently release of cytochrome c (Cyt-c) from mitochondria into cytosol. Cyt-c release activates the caspase cascade, promoting apoptosis. Thus, we explored the influence of PRMT6 downregulation on mitochondria-dependent apoptosis. Firstly, we measured the mitochondrial membrane potential by JC-1 fluorescence mitochondrial staining assay. As shown in Fig. 8C, JC-1 aggregates accumulated in the mitochondrial membrane and emitted a strong red fluorescent signal in control HEI-OC1 cells. HEI-OC1 cells exposed to cisplatin resulted in the dissipation of ΔΨm thus showing increased green fluorescence, which indicates the existence of monomeric JC-1 and depolarization of the mitochondrial membrane. However, PRMT6 silenced cells maintained an essential mitochondrial integrity by showing a significant increased red fluorescence. Moreover, the significant decreases of fluorescence intensity of TMRM (Figure 8D) and rhodamine 123 (Figure 8F) in the cisplatin damaged cells also reflected the loss of ΔΨm. In contrast, PRMT6 knockdown by siRNA prevented cisplatin-induced mitochondrial fission (Figure 8D and 8F). Following mitochondrial membrane depolarization, the release of Cyt-c from mitochondria into cytosol is one of the early events during apoptosis. Therefore, we investigated the cellular distribution of Cyt-c by immunostaining assay (Figure 8E). The percentage of cells that released Cyt-c in the cytoplasm was analyzed. At least 200 cells were counted based on three independent experiments. Few Cyt-c in the cytoplasm were found in the control group (1.209 ± 1.959%), whereas an obvious increase of Cyt-c in cytosol occurred in cisplatin damaged cells (61.40 ± 8.198%, *p* < 0.0001). Compared with the cisplatin group, Cyt-c in cytosol in the PRMT6-siRNA-transfected cells significantly decreased (12.24 ± 6.544%, *p* < 0.0001). These results demonstrated that PRMT6 knockdown significantly inhibits cisplatin-induced Cyt-c release into cytoplasm, showing again that PRMT6 knockdown acts on the mitochondria mediated pathway in its otoprotective effect.

## Discussion

Aminoglycosides are used in the treatment of severe or life-threatening infections, and cisplatin is used in the clinical setting to treat many cancers, however, ototoxicity is a serious side effect among patients receiving these drugs. Reducing this toxic effect is a central problem for hearing studies. In the current work, our findings provide *in vitro* and* in vivo* evidence suggesting the benefits of PRMT6 inhibition using pharmacological and genetic interventions, to reduce aminoglycoside and cisplatin-induced ototoxicity, possibly through the preservation of mitochondrial function.

In this study, we first determined the expression of PRMT6 in cochlea and found that neomycin or cisplatin damage increased its expression, providing the precondition for the following investigation of PRMT6 roles in ototoxic drugs-induced toxicity. Next, the effects of PRMT6 on cell apoptosis were assessed in this work. We demonstrated that neomycin and cisplatin increased the cell apoptosis of postnatal mouse cochlear explant cultures in a concentration- and time-dependent manner. Meanwhile, the exposure of cochlear explants to EPZ020411 led to significant protection against neomycin- and cisplatin-induced ototoxicity. In the *in vivo* study, we determined that EPZ020411 pretreatment significantly protected against neomycin- and cisplatin-induced hair cell injury, which attenuated hearing loss in the C57BL/6 mice as evidenced by the reduced ABR thresholds. Note that although PRMT6 inhibition significantly attenuates neomycin or cisplatin-induced ototoxicity, there is still a loss of hair cells and an increase in hearing thresholds compared to undamaged control animals. It is likely that hair cell survival is dependent upon multiple epigenetic mechanisms. For instance, our previous study showed that G9a inhibitor BIX 01294 reduces H3K9me2 levels and prevents hair cells from aminoglycosides-induced death by inhibiting the mitochondrial apoptosis pathway, support the notion that histone modifications are involved in ototoxicity-induced hearing loss 27. We also have previously shown that some LSD1 inhibitors protect against ototoxicity-induced HC death by maintaining H3K4me2 levels 33. Thus, more complete picture of epigenetic and molecular mechanisms should be elucidated in future studies.

Previous studies have reported that apoptotic cell death is the principal mechanism underlying drugs-induced ototoxicity 34, 35. We found that hair cells that were damaged with neomycin or cisplatin showed characteristic apoptotic nuclear morphologies such as condensed chromatin and fragmented DNA. The numbers of cleaved caspase-3/myosin 7a double-positive and TUNEL/myosin 7a double-positive cells were significantly increased in response to damage, indicating that neomycin and cisplatin can cause the death of hair cells mainly by activating apoptosis and causing DNA damage, which is consistent with previous studies. However, very little cleaved caspase-3 and TUNEL staining was observed in explants pretreated with EPZ020411, suggesting that PRMT6 inhibition protects hair cells against neomycin- and cisplatin-mediated ototoxicity through the inhibition of apoptosis.

It is well documented that aminoglycosides and cisplatin appear to cause increased accumulation of ROS, which is known to be the initiating contributor of ototoxic drugs-induced ototoxicity 36, 37. Overproduction of ROS overwhelms the redox balance, triggering mitochondrial depolarization and release of cytochrome* c*, which then activates caspase-3 and ultimately results in apoptosis of hair cells. Thus, the accumulation of ROS has been identified as an important mediator of the toxic effects of aminoglycoside antibiotics and cisplatin as well as noise-induced cochlear hair cell apoptosis 38-40. In the current study, we used the mitochondria-specific ROS indicator MitoSox-Red after neomycin and cisplatin treatment to determine whether EPZ020411 could attenuate neomycin- and cisplatin-induced cell death by decreasing ROS generation. In addition, the protective effect of PRMT6 inhibition was further confirmed in the cochlear hair cell-like HEI-OC1 cell line, a cochlear HC-like cell line. We found that cisplatin exposure caused increased apoptosis and ROS accumulation in HEI-OC1 cells, while the cisplatin injury was significantly reduced by siRNA-mediated PRMT6 knockdown compared to the cisplatin only group, with the decreased number of apoptotic HEI-OC1 cells and decreased ROS level. Moreover, inhibition of PRMT6 mitigated the release of cytochrome *c* and suppressed the level of caspase-3 in HEI-OC1 that were exposed to cisplatin. These findings were in consonance with those from cochlear explants, thereby further strengthening our results *in vitro*.

Several signaling pathways have been implicated in regulating hair cell survival after ototoxic insult, including the JNK, PI3K/AKT, and MEK/ERK pathways. It has been reported that JNK can be activated in response to oxidative stress, which acts as a signal of hair cell damage, and modulation of JNK has been shown to be a promising protective measure in the prevention of hearing loss 41. PI3K/AKT has also recently been shown to be involved in hair cell survival 42. PI3K can be activated by various molecules through cell-surface receptors, and activated PI3K in turn phosphorylates and activates AKT, which serves as an important anti-apoptotic mediator by affecting many downstream effectors of apoptosis 43. Thus, it will be of great interest to address the integration of arginine methylation and divergent signal transduction pathways. Protein arginine methylation, together with phosphorylation, has been shown to be a major post-translational modification that occurs in mammalian cells 44. Recent work reported that H3R2me2a was required for early zebrafish embryogenesis by preventing the activation of the p38/JNK pathway by directly suppressing *gadd45αa* 45. Arginine methylation has recently been implicated in the pathogenesis of several neurodegenerative diseases, such as spinal and bulbar muscular atrophy, caused by polyglutamine expansion in the androgen receptor 46. Arginine methylation can target polyglutamine proteins, and thus arginine plays a role in the pathogenesis of polyglutamine disease by preventing AKT phosphorylation, and treatment with PI3K/AKT signaling inhibitors reduces the effect of arginine methylation inhibition on cell viability. This suggests that arginine methylation might enhance polyglutamine-expanded androgen receptor toxicity by counteracting the phosphorylation of the receptor by AKT. Despite these studies, the underlying mechanisms through which PRMT6 affects hair cell survival in response to ototoxic insults remains unclear. Therefore, in future work we will use appropriate pharmacologic and genetic inhibitory approaches to test if hair cell protection by PRMT6 inhibition is mediated via these signals.

In summary, our findings revealed that inhibition of PRMT6 could attenuate the overproduction of ROS and subsequently suppress the mitochondrial apoptotic pathway and thus protect hair cells from aminoglycoside- and cisplatin-induced ototoxic damage.

## Supplementary Material

Supplementary figures.

## Abbreviations

ABR: auditory brainstem response
Cyt-c: cytochrome c
DMSO: dissolved in dimethyl sulfoxide
HC: hair cell
H3R2me2a: histone H3 at arginine 2
HEI-OC1: House Ear Institute-Organ of Corti 1
IHC: inner hair cells
i.p.: intraperitoneal
JNK: c-Jun N-terminal kinase
MEK/ERK: the mitogen-activated protein kinase kinase/extracellular-signal-regulated kinase
OHC: outer hair cells
P2: postnatal day 2
PBS: phosphate buffered saline
PBST: PBS and permeabilized with 1% Triton X-100 in PBS
PI3K/AKT: phosphoinositide 3-kinase/protein kinase B
PRMTs: protein arginine methyltransferases
PRMT6: protein arginine methyltransferase 6
ROS: reactive oxygen species
TBST: Tris-buffered saline containing 0.1% Tween 20
TUNEL: Terminal deoxynucleotidyl transferase-mediated dUTP nick-end labeling
ΔΨm: mitochondrial membrane potential
