# Supplementary material for: Inhibition of Protein arginine methyltransferase 6 reduces reactive oxygen species production and attenuates aminoglycoside- and cisplatin-induced hair cell death
Source: Theranostics. 2020 Jan 1;10(1):133–50. doi: 10.7150/thno.37362 (PMC6929624; doi:10.7150/thno.37362)
Supplement: Supplementary file 1 — Supplementary figures. [file thnov10p0133s1.pdf]

Inhibition of Protein arginine methyltransferase 6 reduces reactive oxygen species production and attenuates aminoglycoside- and cisplatin-induced hair cell death

Yingzi He[1#], Wen Li[1#], Zhiwei Zheng[2#], Liping Zhao[1], Wenyan Li[1], Yunfeng Wang[1*], Huawei Li[1,3,4,5*]

[1] Department of ENT institute and Otorhinolaryngology, Eye & ENT Hospital, State Key Laboratory of Medical Neurobiology, NHC Key Laboratory of Hearing Medicine Research, Fudan University, Shanghai, 200032, PR China

[2] Department of Otorhinolaryngology Head and Neck Surgery, The First Affiliated Hospital, School of Medicine, Xiamen University, Xiamen, 361003, China

[3] Institutes of Biomedical Sciences, Fudan University, Shanghai, 200032, PR China

[4] Shanghai Engineering Research Centre of Cochlear Implant, Shanghai, 200031, PR China

[5] The Institutes of Brain Science and the Collaborative Innovation Center for Brain Science, Fudan University, Shanghai, 200032, China

[#] Yingzi He, Wen Li and Zhiwei Zheng contributed equally to this work.

Title of running head: Inhibition of PRMT6 protects HC

***To whom correspondence should be addressed:**

Yunfeng Wang, Ph.D
ENT institute and Otorhinolaryngology Department of Affiliated Eye and ENT Hospital, Fudan University
83 Fenyang Road, Shanghai, 200031, China
E-mail: yunfengwang@fudan.edu.cn
Tel: +86-21-64379980; fax: +86-21-64379980

Huawei Li, MD, PhD
ENT institute and Otorhinolaryngology Department of Affiliated Eye and ENT Hospital, Fudan University
83 Fenyang Road, Shanghai, 200031, China
E-mail: hwli@shmu.edu.cn
Tel: +86-21-64377134 669; fax: +86-21-64377151

Supplemental figures and figure legends

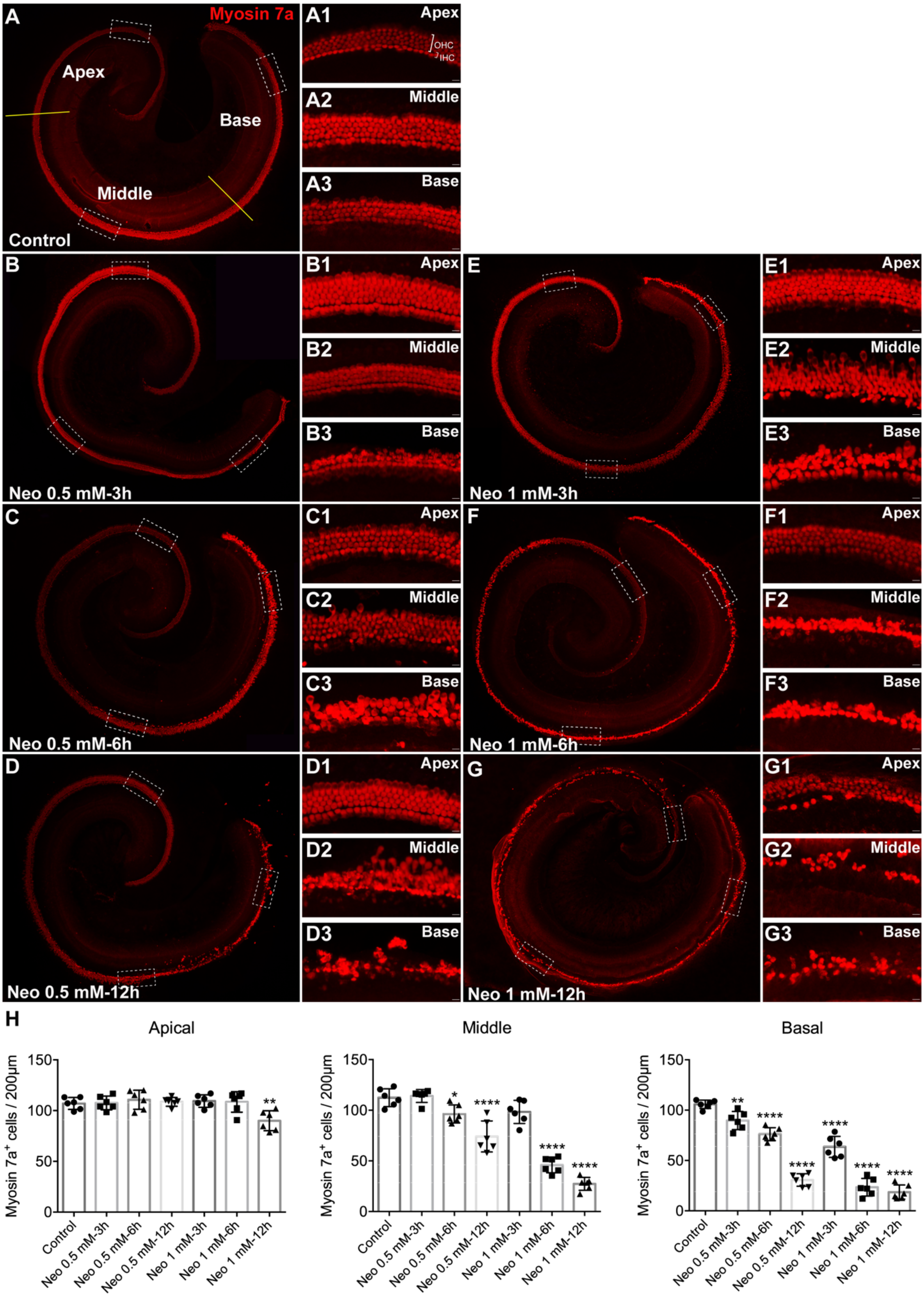

Supplemental Figure 1. Neomycin (Neo) ototoxicity in cochlear explants maintained *in vitro*. (A) Representative immunofluorescence image of HCs labeled with myosin 7a (red) in the cochlear explant. (B-

G) Representative immunofluorescence images of HCs labeled with myosin 7a (red) in the cochlear explants treated with 0.5 and 1 mM neomycin for 3 h, 6 h and 12. Scale bar = 10 µm. (H) Hair cells positive for myosin 7a fluorescence were counted every 200 µm along the apical, middle, and basal regions of the cochlear explants from different groups. Data are presented as the mean ± s.d. one-way ANOVA. *$p < 0.05$, **$p < 0.01$, ****$p < 0.0001$ versus the undamaged group, n = 6 cochlear explants per group. Neo: neomycin; HCs: hair cells.

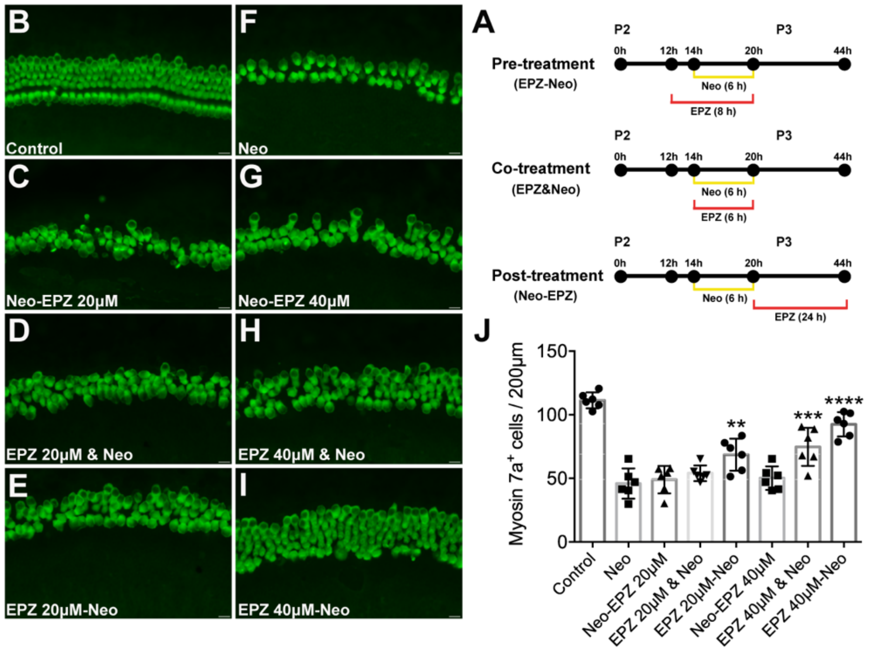

Supplemental Figure 2. Effects of EPZ020411 on hair cell protection against neomycin ototoxicity in cochlear explants maintained *in vitro*. (A) Diagram of neomycin and EPZ020411 administration. (B-I) Representative immunofluorescence images of the middle turns of cochlear explants staining for myosin 7a (green). They were either treated with neomycin alone (Neo), neomycin with EPZ020411 (20 µM, 40 µM) pre-treatment (EPZ 20 µM-Neo, EPZ 40 µM-Neo), neomycin and EPZ020411 co-treatment (EPZ 20 µM & Neo, EPZ 40 µM & Neo), or neomycin with EPZ020411 post treatment (Neo-EPZ 20 µM, Neo-EPZ 40 µM). Scale bars = 10 µm. (J) Quantification of the numbers of myosin 7a-positive cells in middle turns from each group. Data are presented as the mean ± s.d. **$p < 0.01$, ***$p < 0.001$, ****$p < 0.0001$ versus the neomycin (Neo) group, n = 6 cochlear explants per group. Neo: neomycin alone; EPZ 20 µM-Neo: neomycin with 20 µM EPZ020411 pre-treatment; EPZ 40 µM-Neo: neomycin with 40 µM EPZ020411 pre-treatment; EPZ 20 µM & Neo: neomycin and 20 µM EPZ020411 co-treatment; EPZ 40 µM & Neo: neomycin and 40 µM EPZ020411 co-treatment; Neo-EPZ 20 µM: neomycin with 20 µM EPZ020411 post treatment; Neo-EPZ 40 µM: neomycin with 40 µM EPZ020411 post treatment.

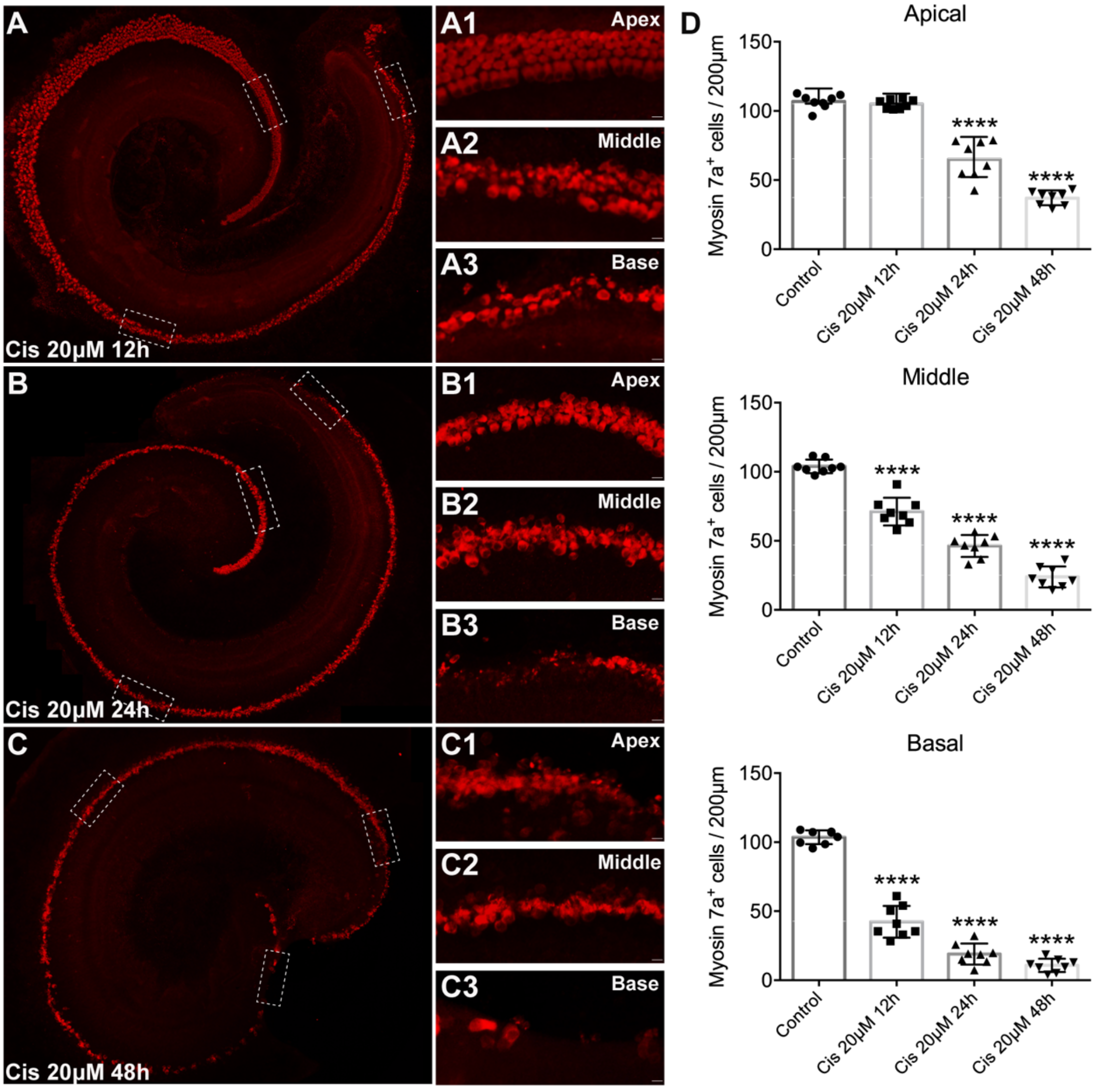

Supplemental Figure 3. Cisplatin (Cis) ototoxicity in cochlear explants maintained *in vitro*. (A-C) Representative immunofluorescence images of HCs labeled with myosin 7a (red) in the cochlear explants treated with 20 μM cisplatin for 12 h, 24 h and 48 h. (D) Quantification of the numbers of myosin 7a-positive cells from each group. Scale bars = 10 μm. The data are presented as the mean ± s.d. one-way ANOVA. ****$p < 0.0001$, n = 8 cochlear explants per group. Cis: cisplatin; HCs: hair cells.

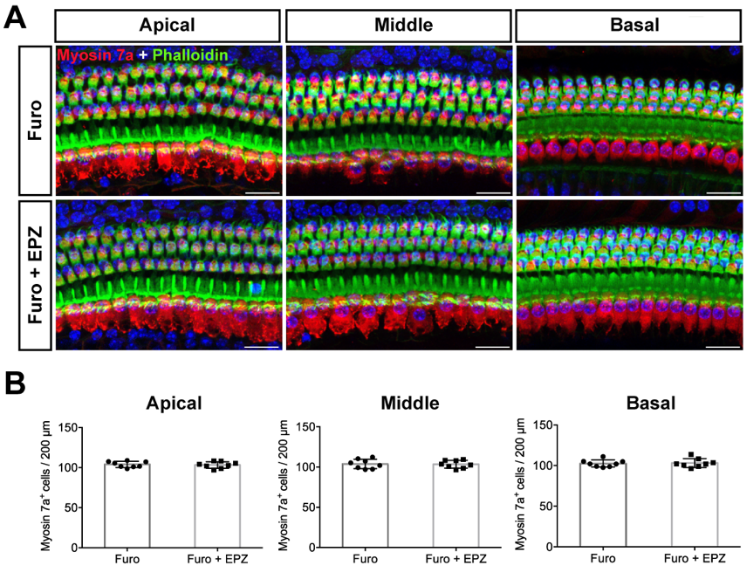

Supplemental Figure 4. Effects of furosemide on hair cells *in vivo*. (A) Representative images of hair cells labeled with myosin 7a (red) + phalloidin (green) in the apical, middle and basal turns of the cochleae from mice received furosemide alone (no neomycin) combined with EPZ020411 (Furo + EPZ) or sterile saline (Furo). Scale bars = 20 µm. (B) Quantification of the numbers of myosin 7a-positive cells. The data are presented as the mean ± s.d. n = 8 cochlear explants per group.

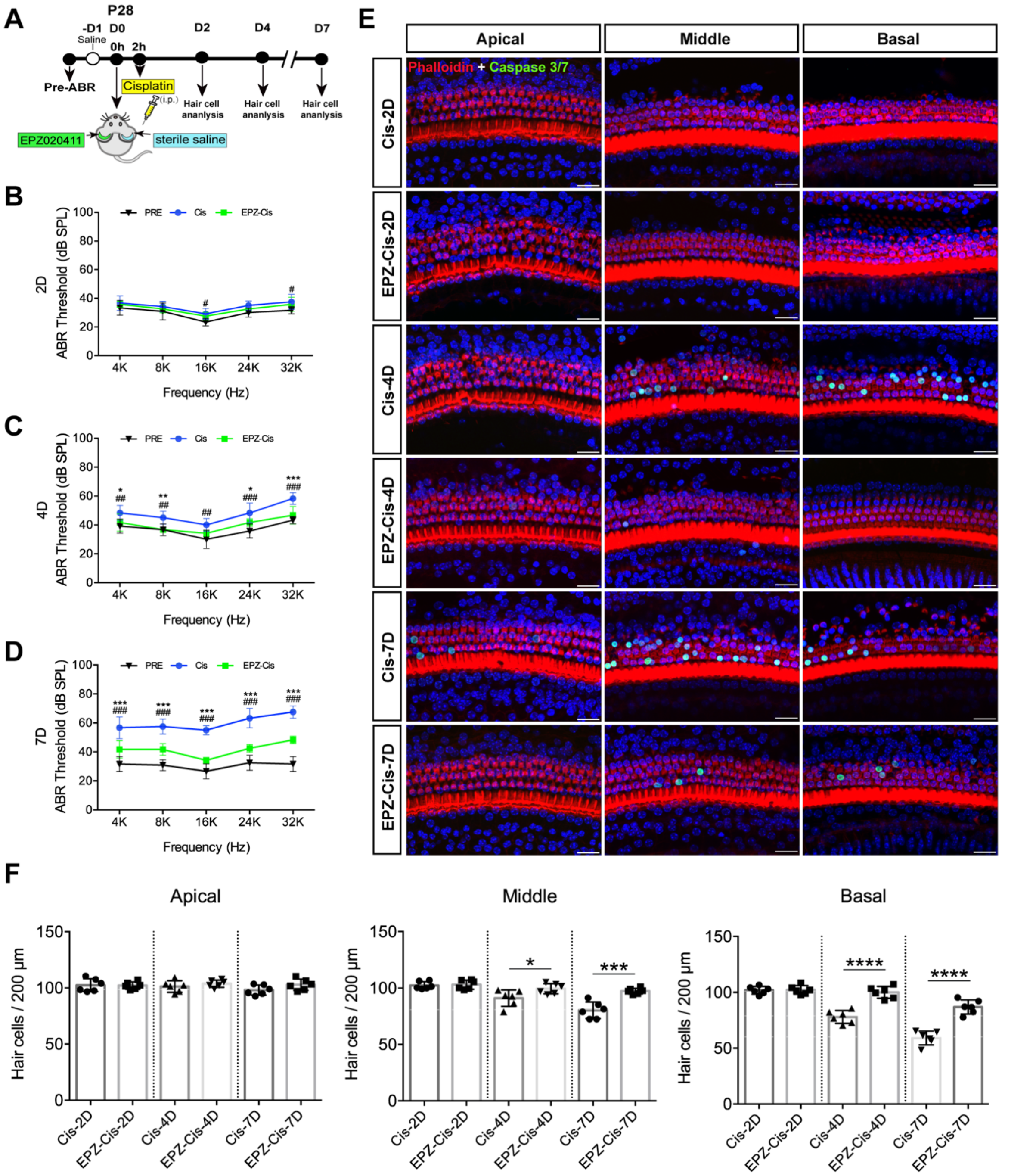

Supplemental Figure 5. *In vivo* time responses of cisplatin in adult mice. (A) Experimental design. (B-D) Comparison of ABR threshold shifts after D2, D4, and D7 for sterile saline and EPZ020411 treatment with cisplatin damage. The data are expressed as the mean ± s.d. $^{\#}p < 0.05$, $^{\#\#}p < 0.01$, $^{\#\#\#}p < 0.001$ versus the control group; $^{*}p < 0.05$, $^{**}p < 0.01$, $^{***}p < 0.001$ versus the EPZ-Cis group, n = 6 cochlear explants per group. (E) Representative images of hair cells labeled with phalloidin (red) and Caspase 3/7 (green) in the apical, middle and basal turns of different groups. Scale bars = 20 μm. (F) Quantification of the numbers of hair cells. The data are presented as the mean ± s.d. $^{*}p < 0.05$, $^{***}p < 0.001$, $^{****}p < 0.0001$, n = 6 cochlear explants per group. Cis: cisplatin; EPZ-Cis: EPZ020411 plus cisplatin; i.p.: intraperitoneal.

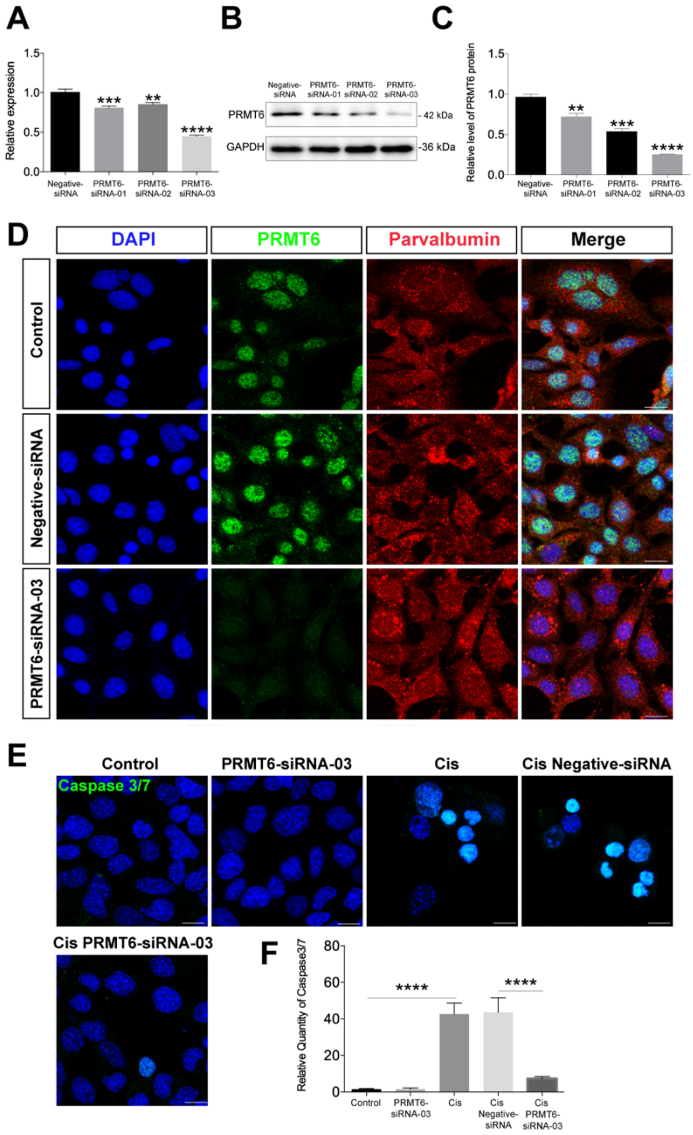

Supplemental Figure 6. Downregulation of PRMT6 by transfection with PRMT6-siRNA. (A) The mRNA levels of PRMT6 in siRNAs transfected HEI-OC1 cells were detected by Q-PCR. Values were normalized relative to the β-actin mRNA levels. Data are expressed as the mean ± s.e.m.. $^{**}p < 0.01$, $^{***}p < 0.001$, $^{****}p < 0.0001$. (B) Immunoblot analyses of PRMT6 expression in HEI-OC1 cells. (C) Semi-quantitative densitometric analyses of PRMT6 was performed using Image J. The protein content was normalized against the corresponding GAPDH level. Data are expressed as the mean ± s.e.m.. $^{**}p < 0.01$, $^{***}p < 0.001$. $^{****}p < 0.0001$. (D) Immunofluorescence staining with PRMT6 (green) and parvalbumin (red) antibodies in cells transfected without or with negative-siRNA and PRMT6-siRNA-03. Scale bar = 10 μm. (E) Representative images of Caspase 3/7 staining in the control, PRMT6-siRNA-03 only, cisplatin only, negative-siRNA and PRMT6-siRNA-03 groups after cisplatin exposure. Scale bars = 10 μm. (F) Quantification of Caspase 3/7–positive cells in five different groups. Data are shown as the mean ± s.e.m.. $^{****}p < 0.0001$.
